# Correcting delayed reporting of COVID‐19 using the generalized‐Dirichlet‐multinomial method

**DOI:** 10.1111/biom.13810

**Published:** 2022-12-27

**Authors:** Oliver Stoner, Alba Halliday, Theo Economou

**Affiliations:** ^1^ School of Mathematics and Statistics University of Glasgow Glasgow UK; ^2^ Climate and Atmosphere Research Centre The Cyprus Institute Aglantzia Cyprus

**Keywords:** Bayesian, forecasting, generalized Dirichlet, notification delay, nowcasting, SARI

## Abstract

The COVID‐19 pandemic has highlighted delayed reporting as a significant impediment to effective disease surveillance and decision‐making. In the absence of timely data, statistical models which account for delays can be adopted to nowcast and forecast cases or deaths. We discuss the four key sources of systematic and random variability in available data for COVID‐19 and other diseases, and critically evaluate current state‐of‐the‐art methods with respect to appropriately separating and capturing this variability. We propose a general hierarchical approach to correcting delayed reporting of COVID‐19 and apply this to daily English hospital deaths, resulting in a flexible prediction tool which could be used to better inform pandemic decision‐making. We compare this approach to competing models with respect to theoretical flexibility and quantitative metrics from a 15‐month rolling prediction experiment imitating a realistic operational scenario. Based on consistent leads in predictive accuracy, bias, and precision, we argue that this approach is an attractive option for correcting delayed reporting of COVID‐19 and future epidemics.

## 1 INTRODUCTION

The coronavirus disease or COVID‐19 is an infectious disease caused by the severe acute respiratory syndrome coronavirus 2 (SARS‐Cov‐2) virus. Like many infectious diseases, data on COVID‐19 cases and deaths are typically subject to delayed reporting, otherwise known as ‘notification delay’. This is when available count data are, for a time, an under‐representation of the truth, owing to flaws or ‘lags’ in the data collection mechanism. In disease surveillance, delays—for example, ones that occur during the transfer of information from local clinics to national surveillance centers—mean that complete and informative counts of new cases or deaths are not immediately available. Often these delays are substantial, so that it can take several weeks or even months for the available data to reach a total reported count.

**FIGURE 1:**
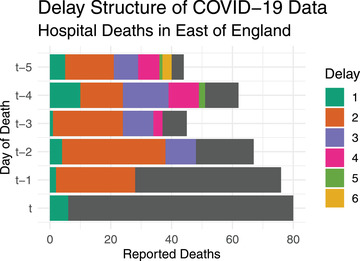
Bar plot of reported COVID‐19 hospital deaths in the East of England region, for the days leading up to and including day *t*, January 1, 2021. The grey bars represent the number of deaths which have not yet been reported as of day t+1 (January 2), while the different colored bars show the number of deaths reported after each day of delay (i.e. published on January 2).This figure appears in color in the electronic version of this paper, and any mention of color refers to that version

From April 2020 until July 2022, the National Health Service for England (NHS England) published daily count data of deaths occurring in hospitals in England of patients who had either tested positive for COVID‐19 or where COVID‐19 was mentioned on their death certificate (NHS England, 2021). Each daily file contained the number of deaths reported in the 24‐h “reporting period” starting 4 pm 2 days prior to publication and ending 4 pm 1 day prior to publication, grouped in time by date of death and in space by seven regions (e.g., London). For example, Figure 1 shows reported COVID‐19 hospital deaths in the East of England in the days leading up to and including day *t*, the January 1, 2021. This figure appears in color in the electronic version of this paper, and any mention of color refers to that version. The colored bars show the partially reported data available at the end of day t+1, the January 2, while the grey bars show the number of deaths that have not been reported as of day t+1. The 24 hour delay between the end of the reporting period and data publication means day t+1 is the earliest any deaths occurring on day *t* will be reported. We refer to this first interval of reporting as ‘the first delay’. In Figure 1, the portion of deaths reported within the first delay is shown in green. For t−1, we have data reported within the first delay (green) as well as ones reported within the ‘second delay’ (orange). We therefore observe one additional portion of deaths—which we call the ‘delayed counts'—for each day we go back into the past.

Significant heterogeneity in the delay mechanism (e.g., in the proportion of deaths reported in the first delay) makes it challenging to draw conclusions about the total counts in a timely manner. For example, fewer deaths occurring on day t−1 were reported in the first 2 delays (green plus orange) than for t−2 (Figure 1). For a practitioner analyzing the data published at the end of day t+1, there is no clear sign from the available reported counts that the total deaths occurring on day t−1 is in fact larger. Delayed reporting can therefore make it difficult to confidently detect a disease outbreak within a time frame during which interventions are most effective. For COVID‐19, failure to tackle local or regional outbreaks in a timely manner carries the risk of loss of life, while unnecessary interventions can also be costly for the local economy or other aspects of population well‐being.

For effective disease surveillance, we need to correct the delayed reporting and in doing so predict total counts (e.g., the number of deaths) for both recent days (nowcasting) and future days (forecasting), based on any available partial counts and potentially on any historical total counts which have now been fully observed. This necessitates careful treatment of the variability associated with both the total counts and the delayed reporting, beyond the capability of standard statistical methods. Here, we demonstrate that a general generalized‐Dirichlet‐multinomial (GDM) hierarchical framework published in the year prior to the pandemic can address the challenges associated with correcting delayed reporting of COVID‐19, resulting in a versatile operational tool for decision‐makers. In recent years, several compelling methods have been proposed for correcting delayed reporting, and we aim to show that the GDM approach can yield considerably more accurate and precise predictions, to better inform pandemic decision‐making. At the same time, the generality of the GDM framework enables novel insights into the structure of the reporting delay, for example, weekly cycles, which can help inform improvements to reporting processes.

The paper is structured as follows. In Section 2, we discuss the need to consider different sources of variability in COVID‐19 data suffering from delayed reporting and use this as a principled basis for comparing current approaches; in Section 3, we present the general framework for correcting delayed reporting in COVID‐19 data; in Section 4, we apply this framework to counts of hospital deaths from COVID‐19 in regions of England and present a 15‐month rolling prediction experiment to illustrate the GDM method's operational effectiveness in comparison with other approaches. Finally, we conclude with a critical discussion of our approach and avenues for future research in Section 5.

Accompanying the paper is a substantial Web Appendix structured as follows: in Web Appendix A, we apply our approach to severe acute respiratory infection (SARI) data from Brazil, demonstrating applicability to general disease surveillance data; in Web Appendix B, we illustrate the COVID‐19 data structure using examples; in Web Appendix C, we present a simulation experiment that assesses the ability of the framework (i) to appropriately infer covariate effects on disease incidence and reporting delays, and (ii) to capture unknown delay variance structures; in Web Appendix D, we present the mathematical formulation of competing models appearing in Section 4; in Web Appendix E, we explain and study the use of moving data windows to improve computational feasibility; and in Web Appendix F, we discuss how under‐reporting in the overall counts can be allowed for.

## 2 BACKGROUND

**FIGURE 2:**
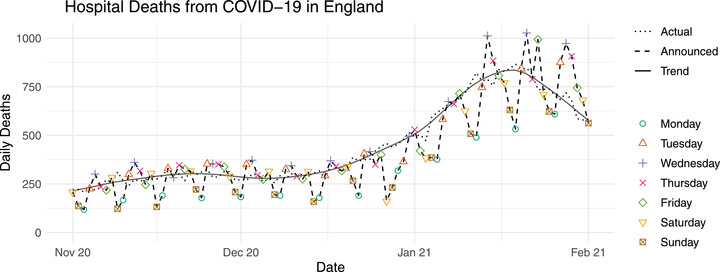
Scatter plot of daily hospital deaths in England. Dashed line and points: the number of deaths reported on each day (announced deaths), by publication date. Different shapes and colors represent the day of the week. Dotted line: the number of actual deaths on each day (*y*). Solid line: smooth trend of the actual deaths. This figure appears in color in the electronic version of this paper, and any mention of color refers to that version

We begin by introducing some notation. Let $y_{t}$ be the number of COVID‐19 deaths or cases occurring on a given day *t*, and let $z_{t,d}$ be the portion of $y_{t}$ observed within d=1,⋯,D delays, so that $\sum_{d=1}^{D}z_{t,d}=y_{t}$. More generally, $y_{t}$ is a count and *t* is the associated time step (e.g., weekly dengue cases). To better understand existing modeling approaches, it is instructive to appreciate the different sources of variability which might be present in data relating to COVID‐19 but also other diseases. Suppose we arrange $z_{t,d}$ into a matrix $z_{t,t^{\prime }}^{\prime }$ of counts where the rows are the date of death *t* and the columns are the date $z_{t,d}$ was first reported, where $t^{\prime }=t+d$ (see Table 1 of the Web Appendix). Taking the sum across columns for each row results in the total deaths occurring on each day $y_{t}$, which we call the ‘actual’ deaths. The $y_{t}$ are of course unknown on day *t* due to delayed reporting. Alternatively, taking the sum across rows for each column results in the total deaths *reported* (rather than occurred) on each day, which we call the ‘announced’ deaths in the case of COVID‐19 mortality. The announced deaths are, by definition, known for days up to and including the most recent date of publication, but tend to consist of deaths which occurred days ago. Figure 2 shows both the ‘actual’ (dotted line) and ‘announced’ (dashed line) in‐hospital deaths from COVID‐19 on each day in England between the October 1, 2020 and the March 1, 2021. This period broadly captures the second wave of COVID‐19, which brought more than 800 deaths per day at its peak. Both the actual and announced deaths follow a clear trend, where daily fatalities reached an initial maximum in November–December before later accelerating to a more severe peak in January. This trend, illustrated by the solid line, is what we call the ‘systematic variability’ in $y_{t}$, which will vary regionally, for example, due to different population sizes, population densities or time since the disease took hold of the region. The day‐to‐day fluctuation about the smooth curve is what we call the ‘random variability’ of $y_{t}$.

**TABLE 1:** Nowcasting performance metrics of competing models in the COVID‐19 rolling prediction experiment: mean average error (MAE); root‐mean squared error (RMSE), bias, mean 95% prediction interval width (PIW), 95% prediction interval coverage (coverage)

	East of England						London				
	MAE	RMSE	Bias	PIW	Coverage		MAE	RMSE	Bias	PIW	Coverage
GDM‐S	2.8	4.6	−0.4	16	0.97	GDM‐S	4.6	9	−0.2	20	0.95
GDM‐H	3.2	5.9	0.1	17	0.98	INLA	4.8	9.9	‐0.6	25	0.97
NB‐S	3.2	5.3	−0.4	21	0.98	NB‐S	4.8	9.7	−0.2	26	0.96
NobBS‐14	4	7.5	−0.8	33	0.99	GDM‐H	5.1	12	0.4	20	0.94
INLA	4	7.7	−1.1	21	0.97	NobBS−14	5.4	12	−0.6	34	0.98
NobBS	5.1	9.4	−2.4	25	0.93	NobBS	5.4	11	−1.7	32	0.96
	**Midlands**						**Northeast and Yorkshire**				
	MAE	RMSE	Bias	PIW	Coverage		MAE	RMSE	Bias	PIW	Coverage
INLA	4.4	7	−0.3	32	0.99	GDM‐S	3.6	5.5	0.1	20	0.97
GDM‐H	4.5	7.4	0.5	24	0.99	GDM‐H	3.6	5.6	0	19	0.97
GDM‐S	4.5	7.4	0.3	24	0.98	NB‐S	3.8	6.1	−0.6	29	1
NB‐S	4.6	7.8	−0.3	33	0.99	INLA	3.9	5.8	−0.6	24	0.97
NobBS‐14	4.9	7.6	−0.3	40	0.99	NobBS‐14	4	6.2	−0.5	38	0.99
NobBS	6.2	11	−2.4	39	0.96	NobBS	4.9	8.1	−2	31	0.97
	**Northwest**						**Southeast**				
	MAE	RMSE	Bias	PIW	Coverage		MAE	RMSE	Bias	PIW	Coverage
GDM‐H	3.7	5.6	0.3	20	0.99	INLA	3.3	6.4	−0.2	24	0.99
INLA	3.8	5.9	−0.5	26	1	GDM‐H	3.4	6.8	0.4	17	0.99
GDM‐S	4	6.5	0.5	21	0.97	GDM‐S	3.5	6.5	0.7	18	0.98
NobBS	4.8	8	−0.9	36	0.99	NB‐S	3.7	7.5	0.5	23	0.99
NB‐S	4.8	8	0	32	0.99	NobBS	3.7	6.5	−1.3	29	0.94
NobBS‐14	5	8.4	−0.4	40	1	NobBS‐14	3.8	8.5	−0.1	32	0.99
	**Southwest**						**Overall**				
	MAE	RMSE	Bias	PIW	Coverage		MAE	RMSE	Bias	PIW	Coverage
GDM‐H	1.7	3.1	−0.1	10	0.99	GDM‐S	3.5	6.4	0.1	18	0.97
GDM‐S	1.8	3.3	−0.1	10	0.98	GDM‐H	3.6	7	0.2	18	0.98
INLA	1.8	3.1	−0.3	12	0.99	INLA	3.7	6.8	−0.5	23	0.98
NB‐S	1.8	3.6	−0.1	12	0.99	NB‐S	3.8	7.1	−0.1	25	0.99
NobBS‐14	2.2	3.9	−0.2	20	0.99	NobBS‐14	4.2	8	−0.4	34	0.99
NobBS	3	5	−1.7	12	0.88	NobBS	4.7	8.6	−1.8	29	0.95

*Note*: For each region, models are arranged in order of ascending MAEs. GDM‐S means GDM survivor, GDM‐H means GDM hazard, and NB‐S means NB survivor.

In addition to the variability in $y_{t}$, we must furthermore consider variability in the reporting delay, which can also be decomposed into random and systematic. Notice for instance the clear ‘weekly cycle’ in the announced deaths (Figure 2)—also referred to as the ‘weekend effect'—where significantly fewer deaths tend to be announced on Sundays and Mondays. The weekend effect can be explained here by lower levels of administrative staffing at many hospital trusts on Saturday and Sunday. Figure 2 shows some instances of around double the number of deaths being announced on Wednesdays compared to Mondays in the same 7‐day period. In the absence of widespread understanding of delayed‐reporting, such events can generate misguided belief that deaths are ‘surging’ or ‘plummeting’, which highlights the risks of using such data as a raw indicator of the progression of the epidemic. We would also expect systematic between‐region variability in the reporting delay, for example, resource inequality between regions; as well as systematic temporal variability, for example, if reporting efficiency improves over time. From a modeling perspective, failure to take into account this kind of systematic variability in the reporting delay (which in conjunction with systematic variability in $y_{t}$ makes up the overall systematic variability in the delayed counts $z_{t,d}$) means ignoring crucial information when it comes to nowcasting and forecasting.

In summary, attempts to correct for delayed reporting of COVID‐19 should carefully consider the following four sources of variability in the available data:

1. Systematic variability in the total count $y_{t}$ (e.g., exponential growth/decay, seasonal patterns, regional variation).
2. Random variability in $y_{t}$ (e.g., day‐to‐day variation in death count).
3. Systematic variability in the reporting delay (e.g., weekly cycles, improvements in reporting efficiency over time, between‐region differences).
4. Random variability in the reporting delay (e.g., day‐to‐day variation).

The available data at any given time comprise historical (fully) reported counts $y_{t}$ and partial counts $z_{t,d}$ corresponding both to historic $y_{t}$ and to more recent unobserved $y_{t}$. These are the sources of information to be utilized for nowcasting and forecasting and as explained in the following section, the appropriate handling of their respective variability will result in more optimal predictions of current and future counts $y_{t}$.

### 2.1 Review of existing approaches

Stoner and Economou (2020) presented an overview of the well‐established biostatistical literature on correcting reporting delay. We revisit some of that but with a particular focus on utility to COVID‐19 applications: Höhle and an der Heiden (2014) and Salmon et al. (2015) both proposed approaches which combine a Poisson/negative‐binomial model to describe $y_{t}$ with a multinomial model for the partial counts $z_{t,d}|y_{t}$, to describe variability in the delayed reporting. The main strength of these approaches is the intuitive separation of variability (random and systematic) in the total count $y_{t}$ (a and b) from variability in the reporting delay (c and d). Specifically, Höhle and an der Heiden (2014) presented two separate options: (1) the multinomial probabilities are realizations from the generalized‐Dirichlet distribution for each time step, and (2) the multinomial probabilities are modeled with a logistic transformation of potentially informative covariates. The first option offers considerable flexibility to capture different levels of random variability in the reporting delay, but lacks the capability of capturing systematic variability like a weekly‐cycle in reporting performance. This is because the parameters of the generalized‐Dirichlet are not assumed to vary systematically over time or otherwise. The second option allows systematic variability to be captured, at the expense of model fit and non‐optimal predictions in the (very common) situations, where $z_{t,d}|y_{t}$ are over‐dispersed with respect to the multinomial (Stoner & Economou, 2020).

Epidemiological applications (including disease surveillance) often have a spatial dimension (Cabrera & Taylor, 2019) and this is certainly true for COVID‐19, where data are often grouped into geographical units like regions or health authorities. Two existing approaches that deal with spatio‐temporal data are Bastos et al. (2019) and Rotejanaprasert et al. (2020). In both cases, the partial counts $z_{t,d}$ are assumed negative‐binomial in a Bayesian hierarchical framework, where $E\left[z_{t,d}\right]=\mu_{t,d}$ depends on covariates and random effects intended to capture systematic variability in the total count (a)—albeit indirectly through $y_{t}=\sum_{d}z_{t,d}$ – and in the reporting delay (c). This approach, applied to spatio‐temporal SARI data from Brazil (Bastos et al., 2019) and to dengue fever data from Thailand (Rotejanaprasert et al., 2020), is a generalization of older chain‐ladder approaches (e.g., Mack 1993) and is quite flexible, as it can potentially incorporate a wide variety of temporal, spatial, and spatio‐temporal structures. However, the total counts are not explicitly modeled, while the partial counts are assumed independent given covariates and random effects. As such, random variability in the total counts (b) is not necessarily captured well in addition to the added risk of excessive predictive uncertainty when nowcasting and forecasting (Stoner & Economou, 2020). This is in part due to the lack of separation between systematic variability in the total count (a) and the reporting delay (c). A similar approach which partly addresses this separation issue is given by McGough et al. (2020), where the mean of $z_{t,d}$ is defined as $\mu_{t,d}=\beta_{d}\lambda_{t}$. Parameter $\beta_{d}\in \left(0,1\right)$, where $\sum_{d}\beta_{d}=1$, is the proportion expected to be reported with delay *d*, while $\lambda_{t}=E\left[y_{t}\right]$ is effectively the mean of the total count. The proportions $\beta =\{\beta_{d}\}$ are fixed in time, while $\lambda_{t}$ is modeled by random effects at the log‐scale. To account for systematic variation (over time) in the reporting delay (c), the model is applied over a sliding temporal window of fixed length. As such, $\beta_{d}$ is representative of reporting behavior in more recent data. Although this allows flexibility to capture structured temporal variability in the delay, it may result in over‐smoothing of the delay distribution if the window size is too big relative to significant short‐term structured variability in reporting performance (like those exhibited by UK COVID‐19 data, as illustrated later in Figure 4).

**FIGURE 3:**
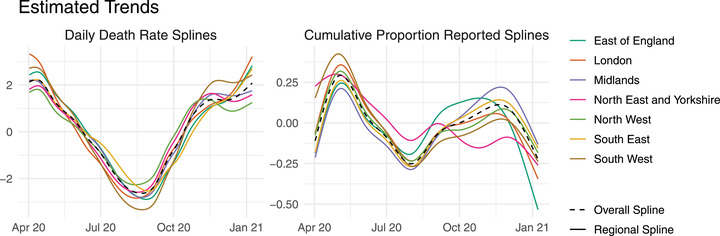
Posterior median spline effects of time on the daily COVID‐19 fatality rate ($\delta_{t,s}$, left) and the cumulative proportion reported ($\beta_{t,s}$, right), for each region. The dashed lines show the overall effects for England, $\alpha_{t}$ (left) and $\xi_{t}$ (right). This figure appears in color in the electronic version of this paper, and any mention of color refers to that version

**FIGURE 4:**
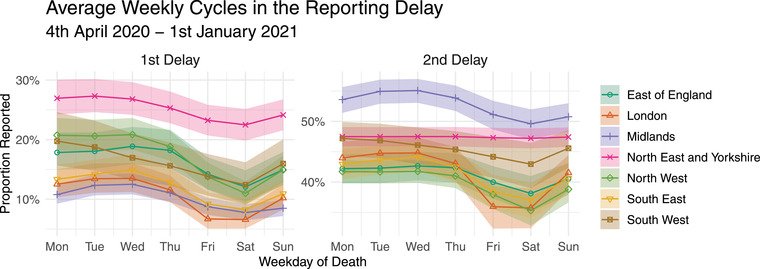
Posterior median (with 95% credible intervals) expected proportion of COVID‐19 deaths reported in the 1st delay (d = 1), left, and in the 2nd delay (d = 2), right, as an overall average from the 4th of April 2020 until the 1st of January 2021. This figure appears in color in the electronic version of this article, and any mention of color refers to that version.

Finally, Stoner & Economou (2020) proposed a general framework for correcting delayed reporting, which utilizes a negative‐binomial model for $y_{t}$ and a GDM model for $z_{t,d}|y_{t}$. Covariates and random effects can be included in the parameters of the GDM, to account for systematic variability in the mean and variance of the reporting delay. The benefit of this approach is that all four sources of variability are accounted for separately using flexible distributions, leading to enhanced interpretability of the model design along with improved prediction performance when nowcasting and forecasting (Stoner & Economou, 2020). In the following sections, we will detail how a spatio‐temporal extension of this framework can be used to correct delayed reporting in COVID‐19 (Section 4) and other disease surveillance data (Web Appendix A).

Existing approaches can be broadly classified into two groups: one where the delayed counts $z_{t,d}$ are modeled marginally without explicitly modeling/using historical information on the totals $y_{t}$, for example, Bastos et al. (2019) and McGough et al. (2020); and another which models the delay counts jointly but also conditionally on $y_{t}$, that is, $z_{t}|y_{t}$, in conjunction with a separate model for $y_{t}$, for example, Stoner & Economou (2020) and Höhle and an der Heiden (2014). We argue that the latter group is better able to explicitly capture (a) and (b) in the model for $y_{t}$, as well as (c) and (d) in the model for $z_{t}|y_{t}$, especially if the model is sufficiently flexible to capture overdispersion relative to the multinomial—like the GDM. Emphasizing that the predictand of interest is the total $y_{t}$, we note that the Bayesian GDM framework produces the predictive distribution $p(y_{t}^{\left(\text{unseen}\right)}|y_{t}^{\left(\text{obs}\right)},z_{t}^{\left(\text{obs}\right)})$, thus utilizing all available information. The ‘marginal’ approaches predict $y_{t}$ indirectly as $\sum_{d}z_{t,d}$, potentially failing to capture random variability in $y_{t}$, due to the absence of an explicit model for $y_{t}$.

In Section 4.5, we apply and compare predictive performance of models based on (i) our GDM approach, (ii) the method in Bastos et al. (2019), and (iii) the method in McGough et al. (2020) to UK COVID‐19 mortality data. In our opinion, these are the three main contenders (in terms of flexibility and practical feasibility) for operational COVID‐19 delay correction.

## 3 MODELING FRAMEWORK

Extending the GDM framework in Stoner & Economou (2020) to include a spatial dimension s∈S (e.g., districts, regions, countries) results in the following model formulation:

(1) $$\begin{array}{ccc} & & y_{t,s}|\lambda_{t,s},\theta_{s}∼\text{Negative-binomial}\left(\lambda_{t,s},\theta_{s}\right);\\ & & \log\left(\lambda_{t,s}\right)=f\left(t,s\right); \end{array}$$

(2) $$\begin{array}{ccc} z_{t,s}|y_{t,s},\nu_{t,s},\phi_{t,s} & ∼ & \text{GDM}(\nu_{t,s},\phi_{t,s},y_{t,s}). \end{array}$$

Systematic spatio‐temporal variability in the total counts $y_{t,s}$ is captured by the general function f(t,s), which may include an offset (e.g., population), covariates or random effects. Variability in the delay mechanism is modeled by the GDM distribution, a multinomial mixture whose vector of probabilities has a generalized‐Dirichlet distribution (Wong, 1998). The use of the GDM for modeling the partial counts, instead of the multinomial, affords a great deal of extra flexibility in accounting for over‐dispersion in the random variability of the reporting delay (d) which improves nowcasting efforts—and in capturing unusual covariance structures in the partial counts (Stoner & Economou, 2020). Here, we choose to parameterize the GDM in terms of $\nu_{t,s}=\left(\nu_{t,s,1},\cdots ,\nu_{t,s,D}\right)$ and $\phi_{t,s}=\left(\phi_{t,s,1},\cdots ,\phi_{t,s,D}\right)$. These are respectively the mean and dispersion parameters of the beta‐binomial conditional models for each partial count:

(3) $$\begin{array}{ccc} & & z_{t,s,d}|z_{t,s,-d},y_{t,s},\nu_{t,s,d},\phi_{t,s,d}∼\text{Beta-binomial}\\ & & \;\;\;\left(\nu_{t,s,d},\phi_{t,s,d},n_{t,s,d}=y_{t,s}-\sum_{j<d}z_{t,s,j}\right). \end{array}$$

Parameter $\nu_{t,s,d}$ (the relative mean) is therefore the proportion of the yet‐to‐be unreported part of $y_{t,s}$ which is expected to be reported at delay *d*. In Stoner & Economou (2020), two options were suggested for modeling the relative means $\nu_{t,s,d}$. In the first (named the Hazard variant), they are modeled directly with a logit link, so that:

(4) $$\log\left(\frac{\nu_{t,s,d}}{1-\nu_{t,s,d}}\right)=g\left(t,s,d\right),$$

for some general function g(t,s,d). In the second (the survivor variant), a model is constructed for $S_{t,s,d}$, the expected cumulative proportion reported before and including delay *d*:

(5) $$\begin{array}{c} \text{probit}\left(S_{t,s,d}\right)=g\left(t,s,d\right). \end{array}$$

The relative means are then easily derived as $\nu_{t,s,d}=\left(S_{t,s,d}-S_{t,s,d-1}\right)/\left(1-S_{t,s,d-1}\right)$. Stoner & Economou (2020) argued that it is more intuitive to consider models for the cumulative proportion of $y_{t,s}$ reported by delay *d*, than to consider models for the expected proportion of $y_{t,s}$ reported at delay *d* out of those not already reported by delay d−1, and so advocate for the survivor variant over the hazard variant. Here, we take a more nuanced view that both variants have merits. Using the hazard variant, it is more straightforward to specify flexible models that capture more complex delay distributions, while for the survivor variant g(t,s,d) must be monotonically increasing in *d*. This constrains the choice of functions and may result in less efficient sampling of related parameters. Meanwhile, using the survivor variant it is more straightforward to specify simple models for the mean delay distribution, which if appropriate may result in more reliable predictive performance. Because we implement the GDM framework using flexible Markov chain Monte Carlo (MCMC) software (Section 4.3), it is possible to try both variants, as well as a variety of choices for f(t,s) and g(t,s,d) (e.g., random walks, autoregressive terms, Gaussian processes) to capture structured variability in the total counts (deaths) and the reporting delay. In the application to COVID‐19 mortality data in Section 4, we opt for a GDM Survivor model where f(·) and g(·) consist of regionally‐structured penalized regression splines, to capture spatio‐temporal variability. Later in Section 4.5, we employ models based on both variants in a rolling prediction experiment.

## 4 APPLICATION TO COVID‐19 DEATHS

Where testing is not widely available, deaths can be more reliable for surveillance than case counts, as those who have died are more likely to have been hospitalized and therefore tested (Lu et al., 2021). In the UK, for instance, testing was not available for community cases early on in the pandemic—reflecting infrastructure limitations (Iacobucci, 2020)—leading to severe under‐reporting. In subsequent months, community testing was available but not evenly distributed in space and time. These issues highlight the importance of COVID‐19 deaths as a key indicator for informing government decision‐making (Seaman et al., 2022).

### 4.1 Data

Recall from Section 1 that the National Health Service for England (NHS England) publishes daily count data of deaths occurring in hospitals in England. These counts were of patients who had either tested positive for COVID‐19 or where COVID‐19 was mentioned on their death certificate (NHS England, 2021). Focusing on data for an individual region *s* for the moment, we first assemble published files into a matrix of counts $z_{t,t^{\prime }}^{\prime }$, where rows correspond to the dates of death *t* and columns correspond to the dates data are published $t^{\prime }$. This is the same matrix described in Section 2, from which we can easily derive the daily ‘announced’ deaths and the daily ‘actual’ deaths. For modeling, we can organize the columns according to the reporting delay $d=t^{\prime }-t$ between the date of death *t* and the data publication dates $t^{\prime }$ (see Table 2 of the Web Appendix). Recall that data for each 24‐h reporting period are published 1 day after the period ends, meaning that d≥1 day. This results in the matrix $z_{t,d}$, which can be combined across regions into a 3D array of counts $z_{t,s,d}$.

As with other approaches (e.g., McGough et al. 2020), the total counts must be assumed fully reported after a specified delay cut‐off, $D_{\max}$. Resulting predictions of $y_{t,s}$ therefore correspond to the number of cases/deaths reported $D_{\max}$ days after the actual day of death. If only a low proportion (e.g., <50% of $y_{t,s}$) is reported after the first $D_{\max}$ delays, nowcasts, and forecasts will not offer a complete picture of ongoing or upcoming outbreaks to decision‐makers. If $D_{\max}$ is needlessly high, then more data on totals $y_{t,s}$ will be unknown and thus require sampling during model fitting, increasing the complexity of the model and potentially making the model impractical for frequent use (e.g., daily). Ideally, $D_{\max}$ is chosen to be sufficiently high that on average most of $y_{t,s}$ (e.g., 90%) are reported. The choice of $D_{\max}$ is therefore very application‐dependent but not daunting, because in many applications most of $y_{t,s}$ is reported in the first few delays (i.e., d<10), with less and less reported afterward. For this dataset in the time period April 2, 2020 to October 28, 2021, 93% of all deaths reported within 28 days were reported within 7 days and 97%  were reported within 14 days. Here, we opt for $D_{\max}=14$ days. If no value of $D_{\max}$ is specified, then all $y_{t,s}$ are unknown and the model is non‐identifiable without additional information (e.g., informative prior distributions), similar to the case of correcting under‐reporting (Stoner et al., 2019).

### 4.2 Nested spline model

Stoner & Economou (2020) presented a model for a time series of dengue fever data in Rio de Janeiro, Brazil, where the incidence of the total recorded dengue counts is modeled by the combination of an intercept term, a temporal effect, and a seasonal effect: $f\left(t\right)=\iota +\alpha_{t}+\eta_{t}$. The temporal ($\alpha_{t}$) and seasonal ($\eta_{t}$) effects were defined using penalized cubic splines, and set up using the jagam function from the mgcv package for the R programming language (Wood, 2016). This was shown to be a very flexible model in capturing smooth temporal and seasonal variation, so we also consider it here to describe the time series of COVID‐19 deaths counts for any individual region, though dropping the seasonal component (as we have only a few months of data). To capture spatio‐temporal variability, we extend this to include spatially‐varying intercept and temporal effects:

(6) $$\begin{array}{c} f\left(t,s\right)=\iota_{s}+\delta_{t,s}, \end{array}$$

with $\iota_{s}$ assigned a non‐informative Normal(0, 10^2^) prior distribution and $\delta_{t,s}$ characterized using penalized cubic splines of time for each region, defined by $\delta_{t,s}=X_{t}\kappa_{s}^{\left(\delta \right)}$. Here, $X_{t}$ is a model matrix of the basis functions evaluated at each time point, and $\kappa_{s}^{\left(\delta \right)}$ is a vector of coefficients. To penalize the splines for over‐fitting, the coefficients are assigned a multivariate‐normal prior with mean zero and precision matrix $\Omega_{s}^{\left(\delta \right)}=\tau_{s}^{\left(\delta \right)}M^{\left(\delta \right)}$. Matrix $M^{\left(\delta \right)}$ is a known non‐diagonal matrix, scaled by a smoothing (penalty) parameter $\tau_{s}^{\left(\delta \right)}$ (Wood, 2016), so that larger values of $\tau_{s}^{\left(\delta \right)}$ result in a smoother $\delta_{t,s}$ for each *s*.

For applications with a high spatial resolution (e.g., local authorities), incorporating more sophisticated spatio‐temporal structures may enable better understanding of disease spread, allowing resources to be allocated to areas which are likely to be affected in the near future. Additionally, when missing information is not solely due to reporting delays, for example, data loss or national holidays, these structures can allow regions with less data to borrow information from the others. Here, the regions are geographically very large, thus we are more concerned with accounting for similarity in trends between regions—in both the fatality rate and in the reporting delay over time—than with explicitly modeling any space–time interactions.

To achieve this, we can re‐introduce the temporal effect $\alpha_{t}$ and make its (basis function) coefficients the mean of the coefficients for the regional effects $\delta_{t,s}$, that is,

(7) $$\begin{array}{ccc} \alpha_{t} & = & X_{t}\kappa^{\left(\alpha \right)};\\ \kappa^{\left(\alpha \right)} & ∼ & \text{Multivariate-normal}(0,\Omega^{\left(\alpha \right)}=\tau^{\left(\alpha \right)}M^{\left(\alpha \right)});\\ \kappa_{s}^{\left(\delta \right)} & ∼ & \text{Multivariate-normal}(\kappa^{\left(\alpha \right)},\Omega_{s}^{\left(\delta \right)}). \end{array}$$

The function $\alpha_{t}$ therefore captures common temporal variation across all regions (and so can be interpreted as the overall trend in the fatality rate for the whole of England), while the $\delta_{t,s}$ capture regional deviations from these overall trends. The parameter $\tau^{\left(\alpha \right)}$ penalizes the overall (England) effect for smoothness, while the $\tau_{s}^{\left(\delta \right)}$ penalize the smoothness of the regional deviations from the overall effect. The main advantage of using this structure is that $\alpha_{t}$ can capture temporal covariation between regions. This hierarchical pooling is akin to random effect (multi‐level) models that effectively utilize properties of the normal (in this case multivariate normal) distribution, decomposing variability into individual‐level terms centered on overall terms (Gelman & Hill, 2006).

We adopt the same approach when extending the relatively simple (survivor) model used in Stoner & Economou (2020) for the expected cumulative proportion reported at each delay, $g\left(t,d\right)=\psi_{d}+\beta_{t}$, first to include spatial variability and second to account for any weekly cycles (see Figure 2) in the reporting delay:

(8) $$\begin{array}{c} g\left(t,s,d\right)=\psi_{s,d}+\beta_{t,s}+\gamma_{t,s}. \end{array}$$

The ‘delay curve’ effects $\psi_{s,d}$ capture the overall shape of the cumulative proportion reported after each delay and are independent across regions. They are assigned first‐order random walk prior distributions, that is, $\psi_{s,d}∼\text{normal}\left(\psi_{s,d-1},10^{2}\right)$, but truncated such that $\psi_{s,d}>\psi_{s,d-1}$ (since the cumulative proportion should increase with *d*). The temporal effects $\beta_{t,s}$ are penalized cubic splines centered on an overall temporal trend $\xi_{t}$ (as in Equation (7)). Finally, $\gamma_{t,s}$ are independent penalized splines for each region, with a cyclic (periodic) cubic basis over the days of the week to account for systematic variability such as the ‘weekend‐effect’. To summarize, the final model to be fitted is given by Equations (6) and (8), where $\delta_{t,s}$ and $\beta_{t,s}$ are regional splines centered around overall national‐level splines.

### 4.3 Prior distributions and implementation

Prior distributions for other parameters were chosen to constrain the parameter space to reasonable values (relative to the data) but without being overly informative: for the negative‐binomial dispersion parameters $\theta_{s}$ we specified independent Gamma(2,0.02) prior distributions, where the 95% prior credible interval (CI) [12.1,279] covers high levels of over‐dispersion (e.g., $\theta_{s}=20$), while more extreme levels (e.g., $\theta_{s}=10$) are less likely a priori. We also specified Gamma(2,0.02) priors for the beta‐binomial dispersion parameters $\phi_{s,d}$, following the same reasoning. Finally, it can be more interpretable to parameterize the spline precision penalties (e.g., $\tau_{s}^{\left(\delta \right)}$) as standard‐deviation penalties (i.e., $\sigma_{s}^{\left(\delta \right)}=1/{\sqrt{\tau }}_{s}^{\left(\delta \right)}$), so that smaller values for $\sigma_{s}^{\left(\delta \right)}$ correspond to a stricter penalty. For these, we specified positive half‐normal(0,1) prior distributions, meaning smoother functions are more likely a priori.

As discussed in Stoner & Economou (2020), instead of explicitly modeling all available partial counts $z_{t,s,d}$, we can reduce computational complexity by choosing to only explicitly model counts for $d\leq D^{\prime }\leq D_{\max}$. We achieve this by only including the conditional beta‐binomial models for $z_{t,s,d}$ up to $D^{\prime }$, so that the remainder $r_{t,s}=y_{t,s}-\sum_{d=1}^{D^{\prime }}z_{t,s,d}$ is modeled implicitly. The trade‐off associated with this choice is that predictive precision for $y_{t,s,d}$ is reduced, but generally only for past weeks $t\leq t_{0}-D^{\prime }$. Hence, selecting a small $D^{\prime }$ may be considered pragmatic where optimally precise predictions are not needed far into the past. In this experiment, we opt for $D^{\prime }=6$, which we consider sensible in a situation where optimally precise predictions are not needed for 6 days or more into the past.

All code was written in the R programming language. The model was implemented in the nimble package (de Valpine et al., 2017), which facilities highly flexible implementation of Bayesian models using MCMC. We used the Automated Factor Slice Sampler (AFSS), which can efficiently sample vectors of highly correlated parameters (Tibbits et al., 2014), for regional spline coefficients and spline penalty parameters, to reduce the number of MCMC iterations and overall computation time needed for convergence. We ran eight MCMC chains in parallel, with different randomly generated initial values, for 80K iterations, discarding 60K as burn‐in and then thinning by 2. We assessed convergence of the MCMC chains by computing the univariate potential scale reduction factor (PSRF) (Brooks & Gelman, 1998) for all unknown parameters in the model. By convention, starting multiple chains from different initial values, and obtaining a PSRF close to or less than 1.05 for a given parameter is taken to indicate convergence. Here, PSRFs were at most 1.02 across all parameters. Unless otherwise stated, point estimates are posterior medians (50% quantiles of the posterior samples) and 95% posterior credible or prediction intervals are defined by taking the 2.5% and 97.5% quantiles of the samples. Finally, we computed predictions for the whole of England by summing the regional predictions.

### 4.4 Results for January 1, 2021

To illustrate our approach as a tool for real‐time decision‐making, we look at estimates and predictions from the model imagining we are fitting it after 5 pm on January 2, 2021, a point in time where the fatality rate was surging in much of England. We use only data which would have been available then, meaning that the latest date for which we have observed some of the total death count is January 1. In Section 4.5, we then present a rolling prediction experiment to assess nowcasting and forecasting performance when this model and others are employed systematically over a period of 15 months.

The left panel of Figure 3 shows the posterior median splines of time $\delta_{t,s}$ in the mean fatality rate $\lambda_{t,s}$. The dashed line shows the overall effect for England, $\alpha_{t}$. All regions show a peak around the first week of April, before decreasing steadily until reaching apparent minimums around August. Following this, the fatality rate increases sharply in all regions, with some nonlinearity closer to the data cutoff date (January 1). Meanwhile, the right panel of Figure 3 shows the posterior median temporal splines in the probit model for the cumulative proportion reported. Here, higher values mean faster reporting on average. Reporting performance appears to have reached a high point around May 2020, before deteriorating in all regions up to August. Reporting performance then improves in most regions, before declining again up to the data cutoff. A simple relationship between reporting performance and the fatality rate is not immediately obvious when comparing the two.

Combining the cumulative delay effects $\psi_{s,d}$ and the weekly cycle splines $\gamma_{t,s}$, Figure 4 shows the posterior median expected proportion reported in the first delay (d=1), left, and in the second delay (d=2), right, by date of death. Recall that here the first delay means deaths captured by the same 24 h reporting period they occurred in and published the following day at 5 pm, and the second delay means deaths captured within the next reporting period. The two panels show clear evidence of “weekend effects” for most regions, with a noticeably lower proportion of deaths occurring toward the end of the week being captured by the first two delayed counts. In London, for instance, more than twice as many deaths occurring on Wednesday are reported in the first delay interval, on average, compared to deaths occurring on Saturday. The 95% CIs for the weekly cycle splines $\gamma_{t,s}$ on Wednesday and Saturday do not overlap for any region except the southwest, where it is instead the Monday–Saturday difference in reporting which is significant, evidencing the strength of the weekend effect across England.

**FIGURE 5:**
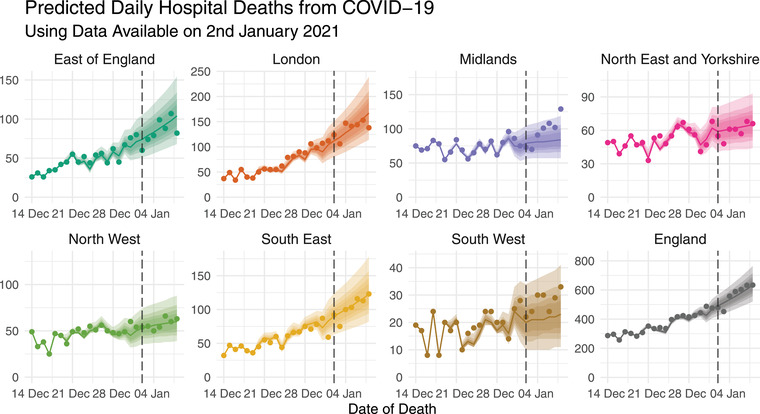
Posterior median nowcasting and forecasting predictions of the total daily deaths $y_{t,s}$ (lines) with up to 95% prediction intervals (shaded areas) for each region, using only data available on January 2 (vertical lines). Points show the total daily deaths reported within 14 days of occurrence (only available with hindsight). This figure appears in color in the electronic version of this paper, and any mention of color refers to that version

Finally, Figure 5 shows nowcasting and forecasting predictions based on data available after 5 pm on January 2, 2021. With hindsight, we can compare predictions to the now fully reported counts to assess performance, plotted as points. Generally, the nowcasting predictions are good; forecasted trends are broadly in line with the data, and uncertainty reflects potential changes in the trend. The next subsection details result from employing this and other approaches repeatedly over a 15 month period.

### 4.5 Rolling prediction experiment

We now aim to assess whether our theoretical arguments in favor of the GDM over competing approaches (Section 2.1) translate into meaningful improvements in prediction performance when systematically applied to real COVID‐19 data in an operational setting. To investigate this, we emulate use of six competing models every 3 days for 15 months (meaning that each model was fitted 153 times). Full details for each competitor model are provided in Web Appendix D, but they can be summarized as follows:

**(1) GDM survivor**: The GDM survivor model described in Section 4.2.
**(2) GDM hazard**: An alternative version of the above model with a GDM hazard formulation for the mean reporting proportions (as described by Equation (4)), including different splines of time and weekly cycles for each delay.
**(3) NB survivor**: A negative‐binomial model for the delayed counts *z*, where the means of *z* are defined by combining exactly the same spline models for the total count and for the cumulative reporting proportions as from the GDM Survivor model in Section 4.2.
**(4) INLA**: An appropriately modified variant of the negative‐binomial model for *z* in Bastos et al. (2019). Notably, we replaced the seasonal component with different weekly cycles for each delay, and we explicitly modeled the remainder term to reduce uncertainty.
**(5) NobBS**: A model for the delayed counts *z* based on the framework proposed in McGough et al. (2020) and implemented using the NobBS package for R.
**(6) NobBS‐14**: A second model based on McGough et al. (2020), where a shorter moving window of 14 days is specified to capture systematic temporal variation in the delay.

So that the comparison can focus primarily on the performance of each modeling framework, rather than any specific spatio‐temporal structures, all models are implemented as independent time series models for each of the seven regions (i.e., nested spline structures are not used in models 1–3). The reason for testing the NB survivor model is to shed some light on the degree to which any differences in performance between the GDM models and the other approaches (i.e., INLA and NobBS) are attributable solely to the use of the full GDM conditional model to appropriately capture variability in the reporting delay. The NB Survivor model is fitted using MCMC because it has a nonlinear mean structure, meaning computation time is comparable to the GDM. For this experiment, we define the “cutoff” date *C* as the day t=C for which we have only observed the first (d=1) portion $z_{C,s,1}$ of deaths $y_{C,s}$ occurring on day *C*. August 1, 2020 is the first cutoff date, while October 31, 2021 is the last. The experiment procedure is as follows:

Step 1: Select an initial data cutoff date *C*.
Step 2: Hold back all partial counts which would have been unavailable then.
Step 3: Fit the models, then predict any partially observed deaths and forecast 7 days ahead.
Step 4: Set the cutoff date C=C+1 and repeat steps 2–4.

**FIGURE 6:**
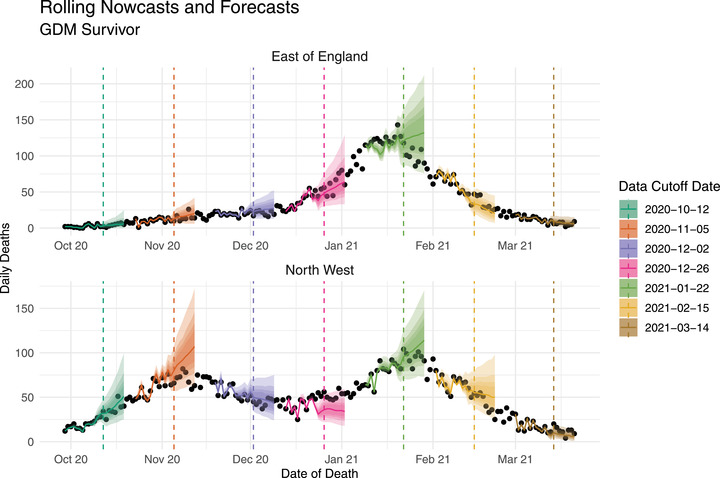
Posterior median nowcasting and forecasting predictions of the total daily deaths $y_{t,s}$ (lines) with up to 95% prediction intervals (shaded areas) for the East of England (top) and Northwest (bottom) regions, from the GDM survivor model. Predictions are shown for seven evenly‐spaced data cutoff dates, where the corresponding cutoff dates are plotted as vertical lines. Points show the total daily deaths reported within 14 days of occurrence. This figure appears in color in the electronic version of this paper, and any mention of color refers to that version

Figure 6 shows predictions from the GDM survivor model in the East of England and Northwest regions, for seven example cutoff dates spaced over part of the experiment period. The figure shows two distinctly shaped time series of daily deaths, with two peaks in the Northwest and one in the East of England. For most of the cutoff dates, the predictions for both regions are satisfying in that the nowcasts (predictions left of the vertical lines) are very close to the true values (the points), while the forecasts generally track the future trends well or otherwise capture them in the 95% prediction intervals. There are naturally some less satisfactory sets of predictions, such as in the Northwest when the cutoff date was December 26 (pink). Here, the predictions appear to carry on the previous slight downward trend, while the points trend upward.

We arranged predictions by the difference, in days, between the date of death each prediction is made for, and the corresponding data cutoff date (t−C)—termed here the “prediction time difference” (PTD). Differences greater than 0 correspond to dates, where none of the deaths were observed yet (i.e., forecasts). Meanwhile, a difference of 0 days or less corresponds to predictions made when at least one part of the total deaths has been observed. Studying prediction performance across the whole range of PTDs is interesting as it shows how the different models cope with different levels of completeness in the available data. Inevitably, though, PTDs close to 0 are most relevant to the goal of correcting reporting delays to provide more accurate and timely disease surveillance, without relying on the ability of specific models to extrapolate into the future appropriately.

We summarize prediction performance by calculating several metrics for each model. The first is the mean average error (MAE) of the posterior median predicted number of deaths occurring on each day, and the second is the root‐mean squared error (RMSE). Both of these quantify how accurate point estimates are, with the RMSE being more sensitive to larger errors. The third is the bias, defined as the mean difference between the median predicted deaths and the observed deaths, which quantifies any systematic over‐ or under‐prediction. The fourth is the mean 95% prediction interval (PI) width for the total number of deaths on each day, which quantifies how precise/uncertain predictions are. The fifth metric is the 95% PI coverage, which checks whether uncertainty is adequately quantified by the model. Here, we use the word “coverage” to describe the proportion of data points contained within their corresponding 95% prediction intervals. Coverage values much less than 0.95 might suggest too few data points are captured by the 95% intervals and the model is *over‐confident*. Conversely, higher coverage values could suggest the predictions display excessive uncertainty. Finally, we computed indicative average daily computation times (see Web Appendix D) to compare the relative practicality of each approach for daily operational use. The GDM survivor approach took 75 min per day, the GDM hazard took 68 min, the NB survivor took 55 min, INLA took 2 min, NobBS took 7 min, and NoBBS‐14 took 1 min.

**FIGURE 7:**
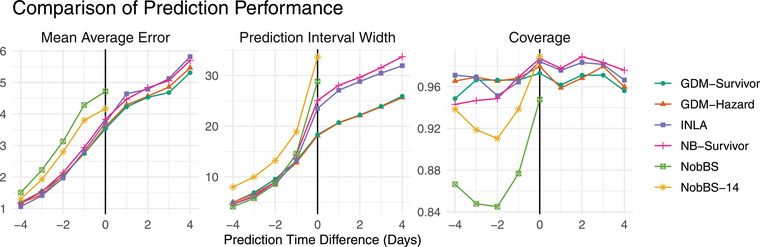
Mean average errors (left), mean 95% prediction interval widths (center), and 95% prediction interval coverage values (right) for daily COVID‐19 deaths in the rolling prediction experiment. Performance metrics are arranged on the *x*‐axis by prediction time difference (PTD), from −4 days up to +4 days, and the different models used to generate predictions are represented by different colors and shapes. This figure appears in color in the electronic version of this paper, and any mention of color refers to that version

We computed these metrics by taking predictions from all predetermined cutoff dates, separately for each PTD. Figure 7 shows the mean average error (left), mean 95% prediction interval width (center), and 95% prediction interval coverage (right) for each model, for PTDs ranging from 4−days up to +4 days. For all models, we can see more accurate and less uncertain predictions for negative differences, because the total counts (deaths) have been more fully observed the further one predicts into the past (and vice versa for forecasting). Meanwhile, if the models are quantifying predictive uncertainty reliably, we should expect high coverage values (>90%) regardless of when we are making predictions for. The coverage values appear quite consistent for all models except the two based on the NobBS method (McGough et al., 2020). When nowcasting and forecasting, the two GDM models offer the lowest MAEs overall, with the NB survivor model and INLA model offering only slightly higher MAEs, and with the highest MAEs coming from the NobBS models. We believe that the weaker performance from the NobBS models in this experiment could largely be because they did not include a weekly cycle in the reporting delay. We believe this because the INLA model had similarly high MAEs before we included different weekly cycles for each delay.

Meanwhile, Table 1 presents the full range of metrics, separately for each region of England and overall, for a PTD of 0 days (nowcasting). Here, the MAE values demonstrate that point estimates from an appropriately designed INLA model (Bastos et al., 2019) can potentially match or sometimes outperform the nowcasting accuracy of those from a more computationally expensive GDM model. However, the mean prediction interval width values from the GDM models show a clear improvement in prediction precision compared to the INLA and NB survivor, which both assume that the parts of the total count reported at each delay are conditionally independent. Table 1 illustrates that GDM leads in prediction interval widths are universal across the seven regions studied, while Figure 7 shows the leads are maintained when forecasting. Considering that the NB survivor and GDM survivor share identical spline models for the mean total deaths and cumulative proportion reported after each delay, the results from this experiment suggest that the hierarchical GDM approach is more effective for quantifying prediction uncertainty in this application.

Similar performance between the two versions of the GDM is reassuring but also interesting, because the GDM hazard version (Web Appendix D) had distinct splines of time and weekly cycles for each delay modeled (d=1,⋯,d=D), meaning we might expect it to better capture complexity in the weekly cycle or changes over time in the reporting delay than the simpler GDM survivor. However, in practice we often see those splines shrinking to 0 for higher delays, as the relative proportions $\nu_{t,s,d}$ become less meaningful.

## 5 DISCUSSION

The COVID‐19 pandemic has highlighted the need to optimally correct delays in disease data for timely mitigation actions. Here, we have critically reviewed the three mainstream approaches to correcting delays, and quantified their respective performance when applied to COVID‐19 mortality data. We have argued that our multivariate approach based on the GDM is theoretically the most advanced in explicitly capturing the different sources of variability in the data. In particular, the separation of systematic variability in the delayed reporting from the systematic variability in the total counts allows novel insights into the structures underpinning each type of variability, for example, weekly cycles in the reporting of COVID‐19. These insights can inform future improvements to reporting timeliness and more reliable conclusions about the progress of the pandemic. In our simulation experiment (Web Appendix C), we demonstrated that the GDM can appropriately separate and capture the effect of covariates imitating real‐world drivers of disease (e.g., vaccination, proliferation of variants) and reporting delays (e.g., staff absence).

Furthermore, of the three current approaches, the GDM is the only one that readily provides predictions of total counts *y* conditional upon *all* available data, that is historic *y* and partial counts z. Indeed, in our realistic rolling COVID‐19 prediction experiment comparing two versions of the GDM against four other models representing the current best‐practice in addressing delayed reporting, the GDM approach was the most optimal in terms of nowcasting accuracy and bias, while demonstrating a clear lead in prediction precision. When nowcasting, the GDM offered a 5%–25% smaller overall RMSE compared to competitors and around a 23%–46% smaller overall mean 95% prediction interval width, while still offering coverage values above 0.95. The GDM leads in prediction precision were consistent across the seven regions of England and were maintained when forecasting.

The GDM framework can accommodate a wide variety of spatial and spatio‐temporal structures in both the model for the total reported counts, and in the model for the delay mechanism. Within this setting, we have developed models based on nested spline structures, to capture similarity of trends between regions. However, for higher spatial resolutions, more sophisticated spatial or spatio‐temporal structures will be necessary, for example, to potentially capture the spread of a disease over time. Moreover, in cases where some regions have a lot of missing data, models with explicit spatial structure may allow for more precise predictions in those regions. Recognizing these points, further development of methods for applications needing more complex spatio‐temporal structures should be a main focus of future work. Finally, applications intended for operational use might also benefit from more complicated mean delay models with delay–time interactions, which are of course possible within the framework proposed here too, for example, using tensor product smooths (Wood, 2006).

Each GDM model of the COVID‐19 deaths took just over 1 h to compile and run (using a moving data window width of 70 days, see Web Appendix E for details). Though our approach is more computationally intensive than competitors, we believe that the run time is reasonable in a daily operational setting, allowing for potential errors and any need to run the MCMC for more iterations for convergence. Indeed, a model based on Stoner & Economou (2020) for nowcasting daily COVID‐19 deaths by age and region in England (Seaman et al., 2022) is used operationally, providing information to the UK Scientific Pandemic Influenza Group on Modelling (SPI‐M) on a weekly basis (MRC Biostatistics Unit, 2020). However, bigger data and model complexity (e.g., COVID‐19 data at hospital trust level) could very easily result in run times in the order of days, so there is a need for either a more efficient implementation of the GDM or a new approach altogether which offers comparable predictive performance to the GDM and improved computational feasibility.

## Supporting information

Web Appendices A–F referenced in Sections 1, 2, 4, and 5 are available with this paper at the Biometrics website on Wiley Online Library. All code and data are available for download as a .zip archive.
Figure 1: Area plot of total recorded severe acute respiratory infection (SARI) cases per 100,000 people, with a different color for each of the 22 health regions Figure 2: Median predicted temporal and seasonal effects on severe acute respiratory infection (SARI) incidence (left and center) and median predicted temporal effect on the cumulative proportion reported (right). Figure 3: Posterior replicates of the sample mean (left) and sample variance (right) of fully observed (*t* ⩽ *t*0‐$D_{\max}$+ 1) total reported number of severe acute respiratory infection (SARI) cases across all 22 health regions. Figure 4: Predicted (median, 50%, 65%, 80%, and 95% prediction intervals) total reported severe acute respiratory infection (SARI) cases for the three most populous health regions Table 1: Invented daily death counts arranged by date of death (rows) and reporting date (columns) Table 2: Invented daily death counts arranged by date of death (rows) and reporting delay *d* (columns) Figure 5: Left: simulated polynomial trends δ*t*,*s* in the mean daily cases Figure 6: Time series of the simulated relative prevalence of two disease variants in the three regions Figure 7: Time series of the mean daily cases $\lambda_{t},s$ (lines) and daily cases $Y_{t},s$ simulated from the negative‐binomial (shapes) Figure 8: Time series of simulated staff absence percentage covariate A *t*,*s* for each of the three regions Figure 9: Time series of the mean cumulative proportion of cases reported, for each of the three regions, by delay Figure 10: Posterior medians (solid lines) and 95% credible intervals (shaded areas) of the polynomial trends δ*t*,*s* in the mean daily cases Figure 11: Posterior densities for the vaccine (left) and variant (right) coefficients (α1 and α2) in the mean daily cases, with dashed lines showing the true values Figure 12: Posterior densities for the time (left), staff absence (center), and variant (right) coefficients in the mean cumulative proportion reported, with dashed lines showing the true values and colors representing each of the three regions. Figure 13: Density estimates of the posterior predictive sample standard deviations of the non‐generalized‐Dirichlet‐multinomial (GDM) delay counts ${\left(z^{(}2\right)}_{t,s,d})$, for the first six delays and each of the three regions Figure 14: Mean average errors (left), mean 95% prediction interval widths (center), and 95% prediction interval coverage values (right) for predicted daily COVID‐19 deaths from generalized‐Dirichlet‐multinomial (GDM) survivor models with different moving window sizes (weeks)

Data S1
