# Supplementary material for: Correcting delayed reporting of COVID‐19 using the generalized‐Dirichlet‐multinomial method
Source: Biometrics. 2022 Dec 27:10.1111/biom.13810. Online ahead of print. doi: 10.1111/biom.13810 (PMC9877609; doi:10.1111/biom.13810)
Supplement: Supplementary file 1 — Web Appendices A–F referenced in Sections 1, 2, 4, and 5 are available with this paper at the Biometrics website on Wiley Online Library. All code and data are available for download as a .zip archive. Figure 1: Area plot of total recorded severe acute respiratory infection (SARI) cases per 100,000 people, with a different color for each of the 22 health regions Figure 2: Median predicted temporal and seasonal effects on severe acute respiratory infection (SARI) incidence (left and center) and median predicted temporal effect on the cumulative proportion reported (right). Figure 3: Posterior replicates of the sample mean (left) and sample variance (right) of fully observed (t ⩽ t0‐Dmax+ 1) total reported number of severe acute respiratory infection (SARI) cases across all 22 health regions. Figure 4: Predicted (median, 50%, 65%, 80%, and 95% prediction intervals) total reported severe acute respiratory infection (SARI) cases for the three most populous health regions Table 1: Invented daily death counts arranged by date of death (rows) and reporting date (columns) Table 2: Invented daily death counts arranged by date of death (rows) and reporting delay d (columns) Figure 5: Left: simulated polynomial trends δt,s in the mean daily cases Figure 6: Time series of the simulated relative prevalence of two disease variants in the three regions Figure 7: Time series of the mean daily cases λt,s (lines) and daily cases Yt,s simulated from the negative‐binomial (shapes) Figure 8: Time series of simulated staff absence percentage covariate A t,s for each of the three regions Figure 9: Time series of the mean cumulative proportion of cases reported, for each of the three regions, by delay Figure 10: Posterior medians (solid lines) and 95% credible intervals (shaded areas) of the polynomial trends δt,s in the mean daily cases Figure 11: Posterior densities for the vaccine (left) and variant (right) coefficients (α1 and α2) in the mean daily cases, with dashed lines showing the [file BIOM-9999-0-s002.pdf]

Supporting Information for "Correcting Delayed Reporting of COVID-19 using the Generalized-Dirichlet-Multinomial Method" by

Oliver Stoner, Alba Halliday and Theo Economou

Web Appendices

Appendix A Severe Acute Respiratory Infection Data

The World Health Organization defines a severe acute respiratory infection (SARI) as an acute respiratory infection where the patient suffers from both a fever measured above 38°C and coughing, where hospitalization is necessary and where the onset of the infection was within the last 10 days (World Health Oganization, 2014). One reason for this classification is to standardise surveillance of influenza-like illnesses, so that seasonal patterns in respiratory virus circulation can be studied and to inform prevention policies (Bastos et al., 2019). As explained in Bastos et al. (2019), lags in data assimilation (from hospitals to local authorities, then to state and national levels) introduce delays in the information on SARI available to public health decision-makers, potentially inhibiting response to influenza outbreaks. In this section we apply our proposed framework to SARI data from Brazil, to illustrate the generality of our approach beyond COVID-19, as well as

to compare a joint (spatio-temporal) model to independent time series models.

A.1 Data

We use data from the Brazilian state of Paraná, which was severely affected by the 2009 H1N1 epidemic compared to other states (Codeço et al., 2012) and continues to have one of the highest rates of SARI incidence (Bastos et al., 2019). Here we consider a much longer time period of 230 weeks (from the start of January 2013 to the end of May 2017), compared to 66 weeks in Bastos et al. (2019), to enable us to draw meaningful conclusions about seasonal variation. The state is divided into $s = \{1, \ldots, 22\}$ health regions and we consider the total count to be fully observed 6 months after occurrence ($D_{max} = 27$). The dimension of the total counts $y_{t,s}$ is therefore 230x22 and the dimension of the partial counts $z_{t,s,d}$ is 230x22x27 (corresponding to over 100k observations). For this application, we imagine that the present-day week, t_0, is week 224 (mid April 2017). We then seek to make predictions for $t_0 = 224$, for previous weeks where the total count is still partially unobserved ($t = \{t_0 - D_{max} + 2, \ldots, t_0 - 1\} = \{199, \ldots, 223\}$) and for the next 6 weeks ($t = \{t_0 + 1, \ldots, t_0 + 6\} = \{225, \ldots, 230\}$).

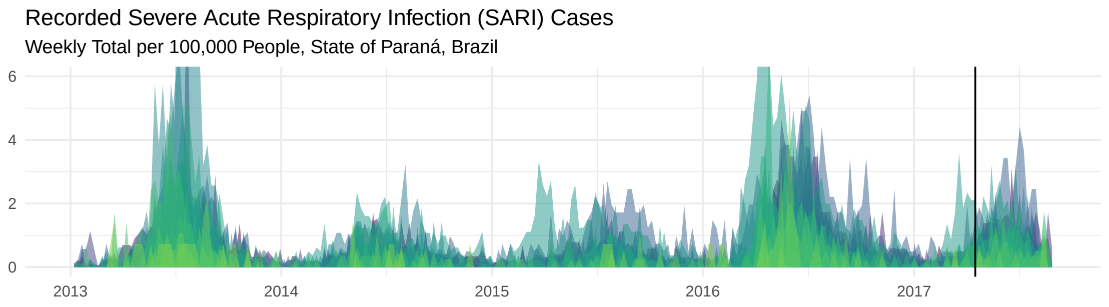

Figure 1: Area plot of total recorded SARI cases per 100,000 people, with a different colour for each of the 22 health regions. The vertical line shows the present week for this experiment $t_0 = 224$.

Figure 1 shows weekly total recorded SARI cases per 100,000 people by region.

The plot shows a clear seasonal cycle across all regions, with outbreaks reaching their peak in April-July, as well as considerable year-to-year variability. There is also some evidence of regional variation in the overall rate – for example, the brightest green region tends to have quite a low rate of cases per 100,000 people, compared to some other regions – as well as regional variation in the seasonal timing of outbreaks. At "present day" $t_0 = 224$, shown by the vertical line, we are in the early stages of the annual influenza outbreak, so forecasting predictions should ideally show an increasing trend in the number of SARI cases.

A.2 Model for SARI cases

In Section 4.2, we introduced a nested spline structure to add a regional dimension to the time series model for dengue fever cases presented in Stoner and Economou (2020). To model the incidence of SARI cases we adopt a near identical approach, making use of spatially-varying intercept, temporal and seasonal effects:

$$f(t, s) = \iota_s + \delta_{t,s} + \xi_{t,s}; \tag{1}$$

Effects $\delta_{t,s}$ and $\xi_{t,s}$ are temporal and seasonal (cyclic) splines, respectively, which vary by region. Figure 1, however, suggests that a large portion of temporal and seasonal variation may be common across all regions. Once again, we can introduce temporal and seasonal effects α_t and η_t, and make their (basis function) coefficients the mean of the coefficients for the regional effects $\delta_{t,s}$ and $\xi_{t,s}$. Similarly, the model for the expected cumulative proportion reported after each delay is characterised by the addition of fixed delay effects $\psi_{s,d}$ which are independent across regions and temporal spline effects $\gamma_{t,s}$:

$$g(t, s, d) = \psi_{s,d} + \gamma_{t,s}. \tag{2}$$

Prior distributions and implementation are the equivalent to the COVID-19 model, detailed in Section 4.3.

A.3 Results

Figure 2 shows median predicted temporal ($\delta_{t,s}$, left) and seasonal ($\xi_{t,s}$, centre) effects on SARI incidence, as well as the temporal effect on the cumulative proportion reported ($\gamma_{t,s}$, right). A different colour is used for each region and the dashed black lines show the median predicted overall effects, α_t, η_t and β_t, respectively. The estimated effects on SARI incidence follow the overall trends quite closely, with only a few deviating substantially. For example, there are noticeable increases in the temporal effect on SARI incidence for almost all regions around mid 2013 and around mid 2016, corresponding to the two largest outbreaks seen in Figure 1. Similarly, all the seasonal effects reflect the increase in SARI incidence leading up to Brazil's winter seen in Figure 1. The effects on the cumulative proportion reported are substantially more variable, suggesting that the delay mechanism may be driven more by local factors compared to SARI incidence.

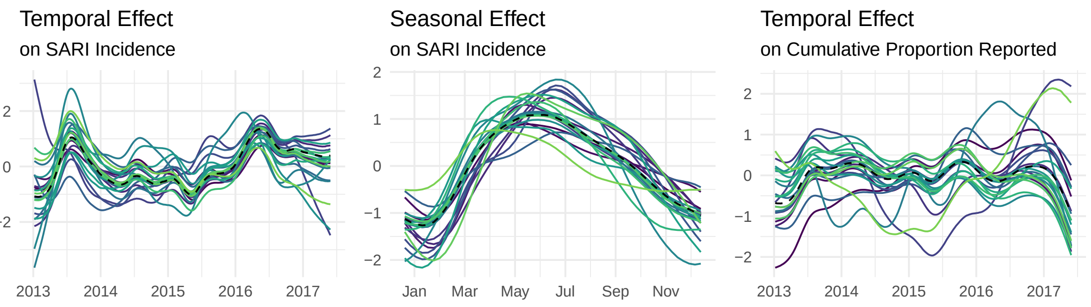

Figure 2: Median predicted temporal and seasonal effects on SARI incidence (left and centre) and median predicted temporal effect on the cumulative proportion reported (right).

Summarising, the 22 health regions of Paraná have a lot in common, in terms of temporal and seasonal variation in SARI incidence. It is worth examining, however, whether anything tangible was gained from modelling the regions simultaneously as opposed to using 22 independent time series models. For instance, modelling the regions independently could impede the model's ability to capture

the variance of the total number of reported cases across all regions. To assess this, we applied 22 independent models where $f(t) = \iota + \delta_t + \xi_t$ and $g(t,d) = \psi_d + \gamma_t$ for each region. We then used posterior predictive checking (Gelman et al., 2014) to see which approach captures the variance of the total better.

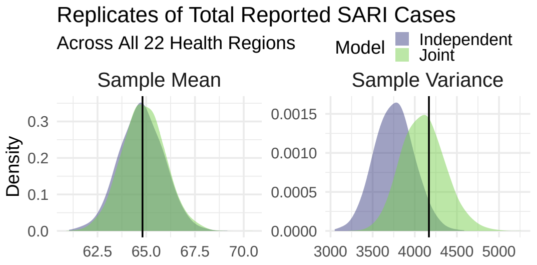

Figure 3: Posterior replicates of the sample mean (left) and sample variance (right) of fully observed ($t \leq t_0 - D_{max} + 1$) total reported number of SARI cases across all 22 health regions. Vertical lines show the corresponding observed statistics.

Figure 3 shows posterior replicates of the sample mean and sample variance of fully observed ($t \leq t_0 - D_{max} + 1$) total reported number of SARI cases across all 22 health regions (i.e. the total for the whole state). Both models perform equally well at capturing the mean number of cases, but notably the joint model is overwhelmingly better at capturing the sample variance compared to the independent models – the observed sample variance is firmly in the centre of the replicate distribution from the joint model. This suggests that the joint model is more appropriate in applications where predictions are also important at a larger geographical scale.

Finally, we can examine the model's ability to nowcast and forecast in this application. Figure 4 shows predicted total reported SARI cases in the three most populous regions of Paraná: Curitiba (left), Londrina (centre) and Maringá (right). Among these three regions, the forecasting predictions when fitted at present week $t_0 = 224$ appear most precise for Curitiba and Londrina, while somewhat over-predicting the number of cases in Maringá. This over-prediction is common across many of the regions, likely owing to the reduced magnitude of

the outbreak compared to the previous year, which the model may detect when fitted further into the outbreak. That said, virtually all of the observations are within the 95% prediction intervals, with precise nowcasting predictions for the present week (shown by the vertical line), which are all within the 50% prediction intervals.

For this experiment, the overall 95% prediction interval coverage was 0.99 for predictions of the total reported count corresponding to previous weeks ($t < t_0$), 1 when forecasting ($t > t_0$) and 1 when nowcasting ($t = t_0$).

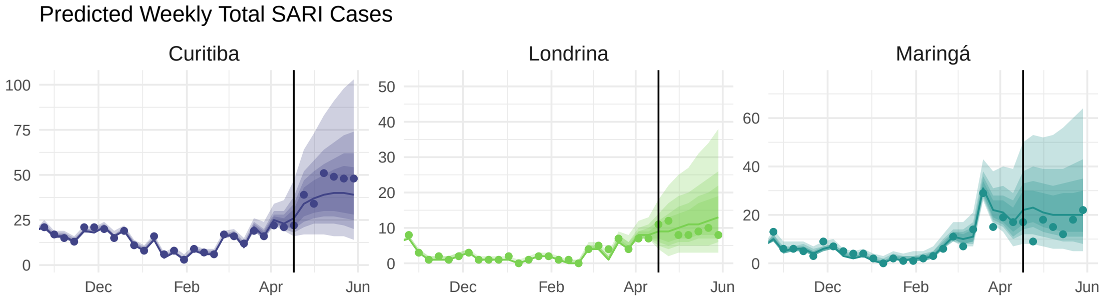

Figure 4: Predicted (median, 50%, 65%, 80%, and 95% prediction intervals) total reported SARI cases for the three most populous health regions. The vertical lines show the present week $t_0 = 224$.

All code and data required to reproduce these results are provided as supplementary material.

Appendix B Illustration of Data Structure

To support understanding of the reporting delay in the COVID-19 hospital deaths application, this section illustrates the data structure. First, recall from Section 1 that a portion of deaths occurring on a given day (e.g. 7th February) will be reported during the same 24-hour reporting period they occurred in and published the following day (8th February) at 5pm. Some more deaths may be reported in

the next 24-hour period, and published the day after that (9th February), and so on until no more deaths are reported. If we compile data files published each day, we can arrange the number of deaths (for each region) by date of death (rows) and reporting date (columns). The below table shows how this might look for some invented values.

		Reporting Date (5pm)			
Date of Death	...	8th Feb	9th Feb	10th Feb	11th Feb
...	...	...	...	...	...
7th Feb	...	12	21	14	5
8th Feb	...		8	15	3
9th Feb	...			35	16
10th Feb	...				54

Table 1: Invented daily death counts arranged by date of death (rows) and reporting date (columns).

Then, assuming a maximum delay of $D_{max} = 4$ days for this example, we can organise the data in terms of the delay between date of occurrence and date of reporting (publication):

Note that there is now a triangular shape of unknown values, shown as "?", which represents the numbers of deaths which are yet to be reported. This is often called the "delay triangle" in the literature. For any date of death where not all delays counts have been observed, the total deaths is also not yet completely observed. The statistical problem is predicting the unknown total deaths, conditional on any partial information for those dates of death, and on any completely observed past total death counts. The final table below shows the same data with modelling notation (where t is the 10th of February):

	Reporting Delay Index				
Date of Death	d=1	d=2	d=3	d=4	**Total Deaths**
...	...	...	...	...	...
7th Feb	12	21	14	5	**52**
8th Feb	8	15	3	?	**?**
9th Feb	35	16	?	?	**?**
10th Feb	54	?	?	?	**?**

Table 2: Invented daily death counts arranged by date of death (rows) and reporting delay d (columns).

	Reporting Delay Index				
Date of Death	d=1	d=2	d=3	d=4	**Total Deaths**
...	...	...	...	...	...
t-3	$z_{t-3,1}$	$z_{t-3,2}$	$z_{t-3,3}$	$z_{t-3,4}$	y_{t-3}
t-2	$z_{t-2,1}$	$z_{t-2,2}$	$z_{t-2,3}$	$z_{t-2,4}$	y_{t-2}
t-1	$z_{t-1,1}$	$z_{t-1,2}$	$z_{t-1,3}$	$z_{t-1,4}$	y_{t-1}
t	$z_{t,1}$	$z_{t,2}$	$z_{t,3}$	$z_{t,4}$	y_t

Table 3: Mathematical notation for daily death counts arranged by date of death (rows) and reporting delay d (columns).

Appendix C Simulation Experiment

In the main article, our assessment of the GDM predominantly focuses on out-of-sample prediction performance, reflecting its intended use as a method for operational nowcasting and forecasting. However, we also claimed that the GDM can provide insights into factors determining the structure of the reporting delay and variability in the total count. We test that here by fitting the GDM to simulated

data, such that inference can be compared against the known effect of covariates.

C.1 Data

The data we simulate are completely synthetic, but are intended to represent a hypothetical disease epidemic, with covariates imitating real-world drivers of change in the disease progression and reporting performance. Note that repeated simulation would be necessary to assess bias, variance, prediction interval coverage etc. Here we simulate one set of data as an example and we use the phrase "true value(s)" to mean the coefficient values chosen in this one example.

Let $y_{t,s}$ be the number of disease cases occurring on day $t \in 1, \ldots, 100$ in region $s \in 1, 2, 3$, and let $z_{t,s,d}$ be the parts of $y_{t,s,d}$ observed at each delay $d \in 1, \ldots, D_{max}$. The experiment focuses on the quality of inference rather than prediction performance, so we assume all $y_{t,s}$ and $z_{t,s,d}$ are known. First, we simulate $y_{t,s}$ from a Negative-Binomial model with mean $\lambda_{t,s}$ and scale parameter θ_s:

$$y_{t,s} \mid \lambda_{t,s}, \theta_s \sim \text{Negative-Binomial}(\lambda_{t,s}, \theta_s); \tag{3}$$

$$\log(\lambda_{t,s}) = \iota_s + \delta_{t,s} + \alpha_1 V_{t,s}^{(*)} + \alpha_2 W_{t,s}^{(*)} \tag{4}$$

The mean number of cases is comprised first of a fourth-order orthogonal polynomials in time $\delta_{t,s}$. The polynomials, shown in the left panel of Figure 5, are distinct for each region to reflect differences in disease progression and non-pharmaceutical interventions. In all 3 regions, the simulated polynomials have an "M" shape, representing two waves of the disease. Then, the mean is also affected by the proportion of the population successfully administered a vaccine in each region, $V_{t,s}$, shown in the right panel of Figure 5. The vaccine becomes available after 40 days, and is then administered at a different rate in each region. After scaling to have 0 mean and standard deviation 1 (let $V_{t,s}^{(*)}$ be the scaled version), our imaginary vaccination covariate has coefficient $\alpha_1 = -1.75$, meaning the case rate reduces

substantially as more people are vaccinated.

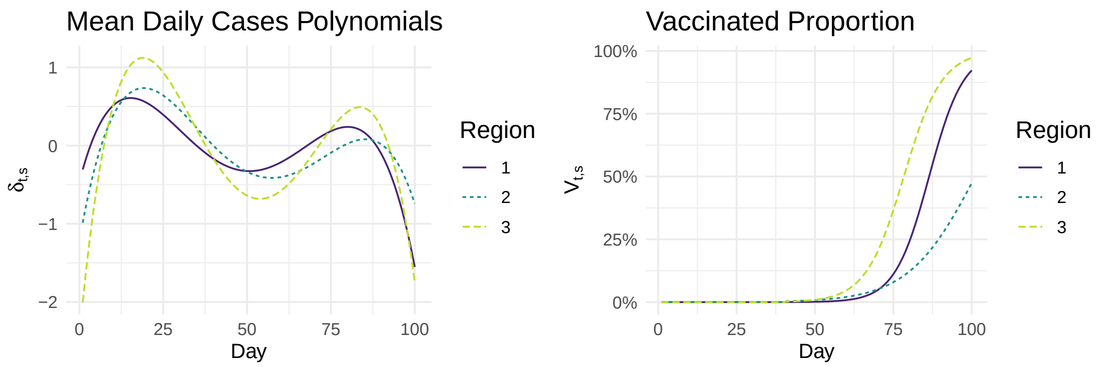

Figure 5: Left: simulated polynomial trends $\delta_{t,s}$ in the mean daily cases. Right: time series of the simulated percentages of the population administered with a vaccine $V_{t,s}$.

Finally, the mean number of cases is affected by the relative prevalence of two variants of the disease, shown in Figure 6. Initially, only Variant 1 is present and then later Variant 2 begins to grow in prevalence at different times and rates depending on the region. Let $W_{t,s}$ be the prevalence of Variant 2, taking values between 0 and 1,and let $W_{t,s}^{(*)}$ be the scaled version. Its associated coefficient is $\alpha_2 = 0.5$, meaning Variant 2 causes more cases than Variant 1. The coefficients of $V_{t,s}^{(*)}$ and $W_{t,s}^{(*)}$ are both assumed constant across regions, meaning we assume the protection of the vaccine and properties of the new variant to be the same across regions.

Combining regional intercept terms ι_s, polynomials $\delta_{t,s}$, the effect of vaccination, and the effect prevalence of variant 2, Figure 7 shows the overall mean daily cases for each region (lines) and then the simulated daily cases $y_{t,s}$ (shapes). The M shape from the polynomials is still visible, but dampened by the effect of the vaccination covariate.

Now, it remains to simulate $z_{t,s,d}$: the parts of $y_{t,s}$ reported at each delay. Here, we simulate the first $D = 7$ delays for modelling. We can ignore the delay structure

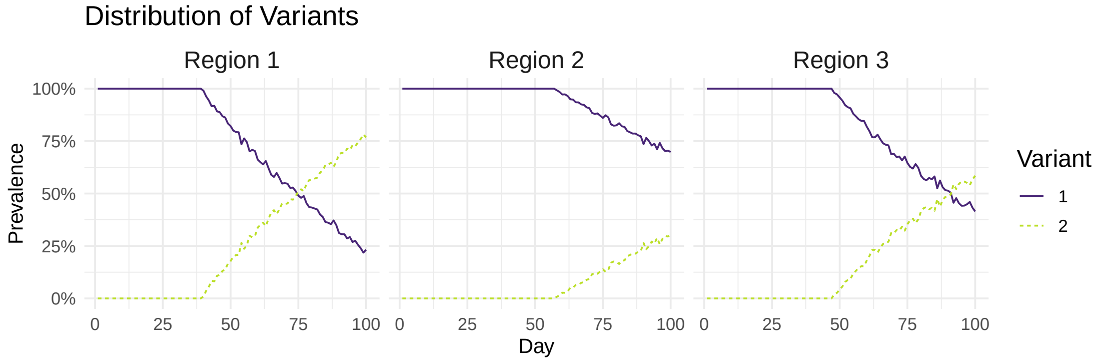

Figure 6: Time series of the simulated relative prevalence of two disease variants in the three regions.

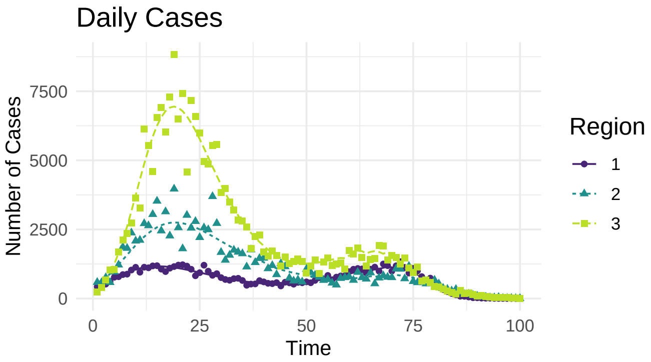

Figure 7: Times series of the mean daily cases $\lambda_{t,s}$ (lines) and daily cases $y_{t,s}$ simulated from the Negative-Binomial (shapes).

of the remainder term because, as mentioned previously, we assume all $z_{t,s,d}$ are observed. To define the mean reporting distribution, we start with a probit model for the expected cumulative proportion reported $S_{t,s,d}$, as in the Survivor variant of the GDM (described in Section 3 of the main article):

$$\text{probit}(S_{t,s,d}) = \psi_{s,d} + \beta_{1,s} X_t^{(*)} + \beta_{2,s} A_{t,s}^{(*)} + \beta_{3,s} \lambda_{t,s}^{(*)}. \tag{5}$$

Temporal variation in $S_{t,s,d}$ is characterised first by monotonically increasing "delay curve" effects $\psi_{s,d}$ and upwards linear trends in (scaled) time $X_t^{(*)}$. We also included staff absence percentage covariates $A_{t,s}$ (Figure 8), simulated as first or-

der autoregressive variables. The coefficients of the scaled absence percentage $A_{t,s}^{(*)}$, $\beta_{2,s}$, are intuitively negative so that a higher percentage of absences slows down reporting.

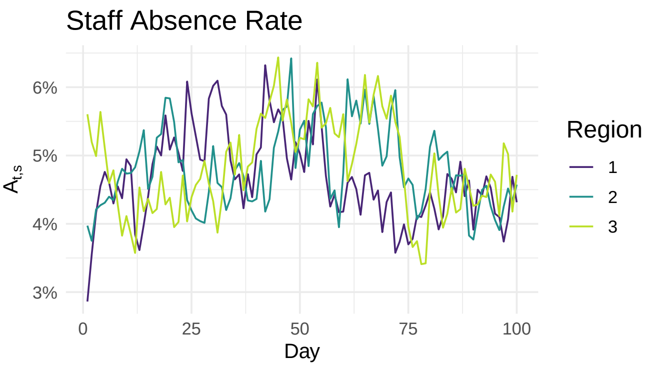

Figure 8: Time series of simulated staff absence percentage covariate $A_{t,s}$ for each of the 3 regions.

Finally, we have also included the scaled mean daily number of cases $\lambda_{t,s}^{(*)}$ as a covariate in the cumulative proportion reported. The coefficients of this effect, $\beta_{3,s}$, are also negative such that, when cases are high, reporting slows down. Inclusion of this covariate is notable because: a) to the best of our knowledge, this is the first attempt (albeit for simulated data) to fit a model for delayed reporting which considers the effect of the same covariates on both the mean total cases/deaths and the reporting delay; and b) effectively including the same covariates in both parts of the GDM hierarchy is more likely to cause inferential problems such as non-identifiability of the covariate coefficients. Combining all of the effects in (5) yields the mean cumulative proportion reported at each delay $S_{t,s,d}$, as shown in Figure 9. Notably, reporting performance visibly slows down in the first 30 days or so as cases surge (as seen in Figure 7). One can imagine this reflecting health systems prioritising treatment over administrative tasks when cases are high.

Using these simulated $S_{t,s,d}$, we then simulated two different sets of delayed counts $z_{t,s,d}$, to be fitted separately. We simulated the first set, $z_{t,s,d}^{(1)}$, from the

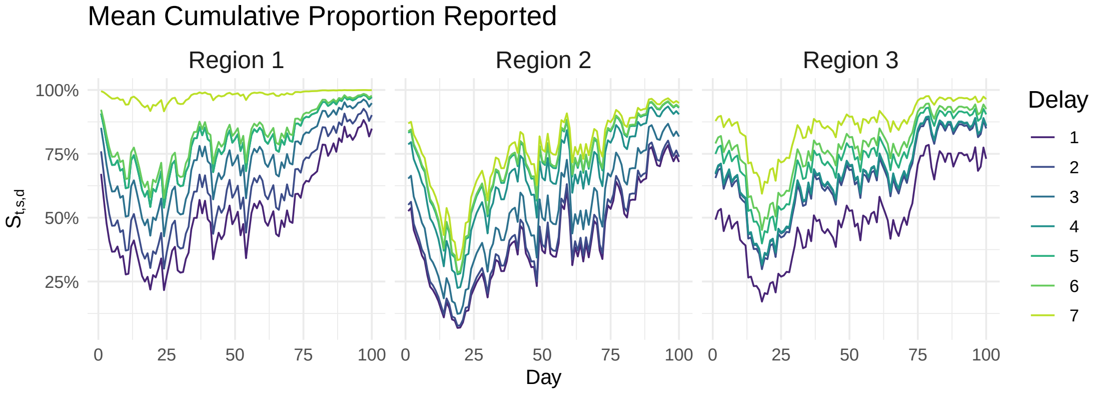

Figure 9: Time series of the mean cumulative proportion of cases reported, for each of the 3 regions, by delay.

Generalised-Dirichlet-Multinomial (GDM) itself:

$$\boldsymbol{z}^{(1)}_{t,s,d} \mid y_{t,s}, \boldsymbol{\nu}_{t,s}, \boldsymbol{\phi}_{s,d} \sim \text{GDM}(\boldsymbol{\nu}_{t,s}, \boldsymbol{\phi}_{s,d}, y_{t,s}), \tag{6}$$

$$\nu_{t,s,d} = (S_{t,s,d} - S_{t,s,d-1})/(1 - S_{t,s,d-1}). \tag{7}$$

This allows us to assess inference when fitting the model to data generated from it. Then, we also simulated a second set, $z^{(2)}_{t,s,d}$, from a series of Binomial-Guassian mixtures. First, we transform the mean proportion reported at each delay $\mu_{t,s,d} = S_{t,s,d} - S_{t,s,d-1}$ into a new set of real number values $u_{t,s,d} \in (-\infty, \infty)$, using a Center Logratio (CLR) transformation:

$$u_{t,s,d} = \log\left(\frac{\mu_{t,s,d}}{\prod_{j=1}^{D_{max}} (\mu_{t,s,j})^{-\frac{1}{D_{max}}}}\right). \tag{8}$$

We then add an i.i.d. Gaussian noise term $\epsilon_{t,s,d} \sim \text{Normal}(0, 0.25^2)$ to each $u_{t,s,d}$ and input this into an inverse CLR transformation to produce a new noisier set of proportions $\mu^{(2)}_{t,s,d}$. A standard deviation of 0.25 was chosen for $\epsilon_{t,s,d}$ so that the variance of $z^{(2)}$ is fairly close to the variance of $z^{(1)}$. From these new (noisier) proportions reported at each delay, we can compute relative proportions $\nu^{(2)}_{t,s,d}$:

$$\nu^{(2)}_{t,s,d} = \mu^{(2)}_{t,s,d} / \sum_{j=1}^{d-1} \mu^{(2)}_{t,s,j}, \tag{9}$$

which can then be used as the mean of Binomial conditional distributions to simulate $z^{(2)}_{t,s,d}$:

$$z^{(2)}_{t,s,d} \sim \text{Binomial}(\nu^{(2)}_{t,s,d}, y_{t,s} - \sum_{j=1}^{d-1} z^{(2)}_{t,s,j}). \tag{10}$$

Fitting a GDM model to $z^{(2)}_{t,s,d}$ then allows us to test the flexibility of the GDM to capture non-GDM variance structures, through posterior predictive checking (Gelman et al., 2014).

C.2 Results

We fit the GDM model outlined in equations (3)-(7) to the simulated two data sets. We use the NIMBLE package (de Valpine et al., 2017) for MCMC and weakly-informative prior distributions (e.g. Normal$(0, 10^2)$ for coefficients), as in the main article.

A key question of interest is whether the known trends and covariate effects in the mean daily cases and in the reporting delay are appropriately captured by the model. To assess this, we should examine outputs from the model fit to $z^{(1)}_{t,s,d}$. We can first look at the polynomial trends in the mean daily cases. Figure 10 shows the posterior median estimates of $\delta_{t,s}$ with 95% credible intervals (CIs). The polynomials are reproduced very closely, with the true values (dashed lines) captured completely by the 95% CIs.

Next, we can look at the coefficients in the mean daily cases for the vaccine (α_1) and for the prevalence of Variant 2 (α_2). Figure 11 shows density estimates of the posterior samples of these two coefficients, with vertical lines representing the true values. In these plots, we are looking to see whether the true values are extreme with respect to their corresponding posterior distributions. Here, the true values for both coefficients are towards the centre of the distributions, indicating the models captures the effect of these covariates well.

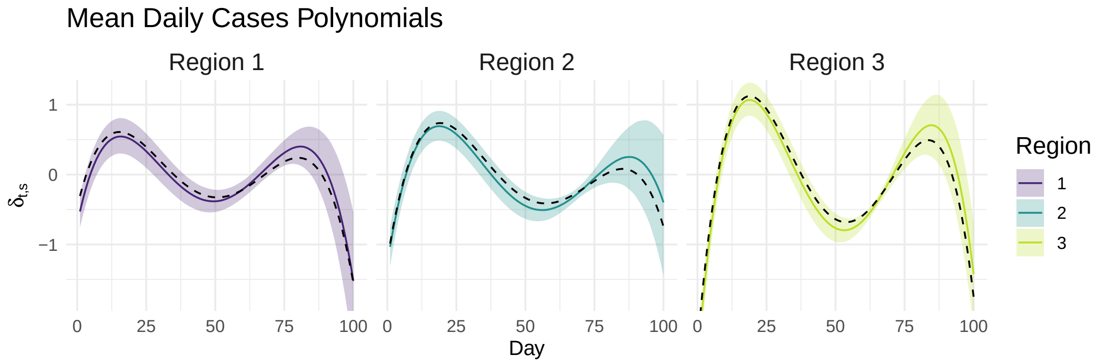

Figure 10: Posterior medians (solid lines) and 95% credible intervals (shaded areas) of the polynomial trends $\delta_{t,s}$ in the mean daily cases. True values are shown as dashed lines.

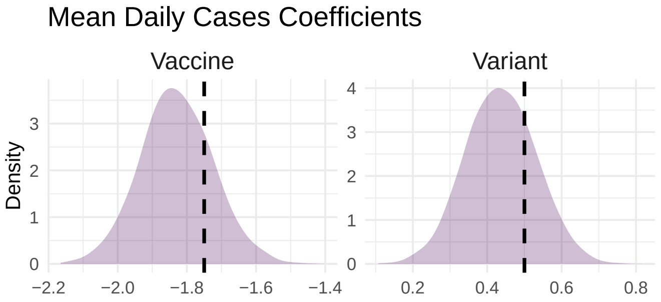

Figure 11: Posterior densities for the vaccine (left) and variant (right) coefficients (α_1 and α_2) in the mean daily cases, with dashed lines showing the true values.

Then, Figure 12 shows the posterior densities for the cumulative proportion reported coefficients of time ($\beta_{1,s}$), staff absence ($\beta_{2,s}$), and the daily case rate ($\beta_{3,s}$), respectively. In most cases, the true values are well within the bulk of the distributions, and after computing 95% CIs we determined that they all contain their corresponding true values.

Finally, we can assess whether the GDM can appropriately capture the variance of the second set of delay counts $z^{(2)}_{t,s,d}$. Recall that these were simulated from Binomial-Gaussian mixture distributions, rather than a GDM. To achieve this, we use the MCMC samples to simulate posterior predictive replicates of the original

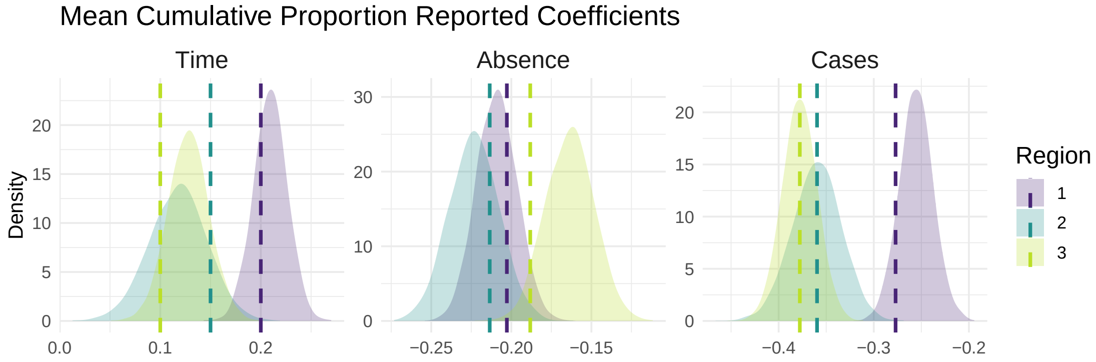

Figure 12: Posterior densities for the time (left), staff absence (centre) and variant (right) coefficients in the mean cumulative proportion reported, with dashed lines showing the true values and colours representing each of the 3 regions.

set of $z^{(2)}_{t,s,d}$, let's call these replicates $\tilde{z}^{(2)}_{t,s,d}$. This results in one set of $\tilde{z}^{(2)}_{t,s,d}$ per saved MCMC iteration. We then compute the sample standard deviation of each set of $\tilde{z}^{(2)}_{t,s,d}$ for each region s and delay d. This results in a distribution of sample standard deviations for each s and d, which we can compare against the corresponding true values from the original $z^{(2)}_{t,s,d}$. Figure 13 shows the posterior predictive sample standard deviations for the first 6 delays. The true values are all within the bulk of the replicate distributions, indicating the model has captured the variances of the delayed counts well despite being generated from a different model.

C.3 Conclusions

Here we simulated a data set of daily disease case counts in 3 regions, with covariates imitating real-world drivers of disease (vaccination, proliferation of different variants) and reporting delays (staff absences, pressure from high case rates). Investigating the latter effect is particularly unusual, because it means we tested a model design that effectively included the same covariates in both the model for the total counts and the model for the reporting delay. Despite this, the GDM was able to reproduce all of the known covariate effects well.

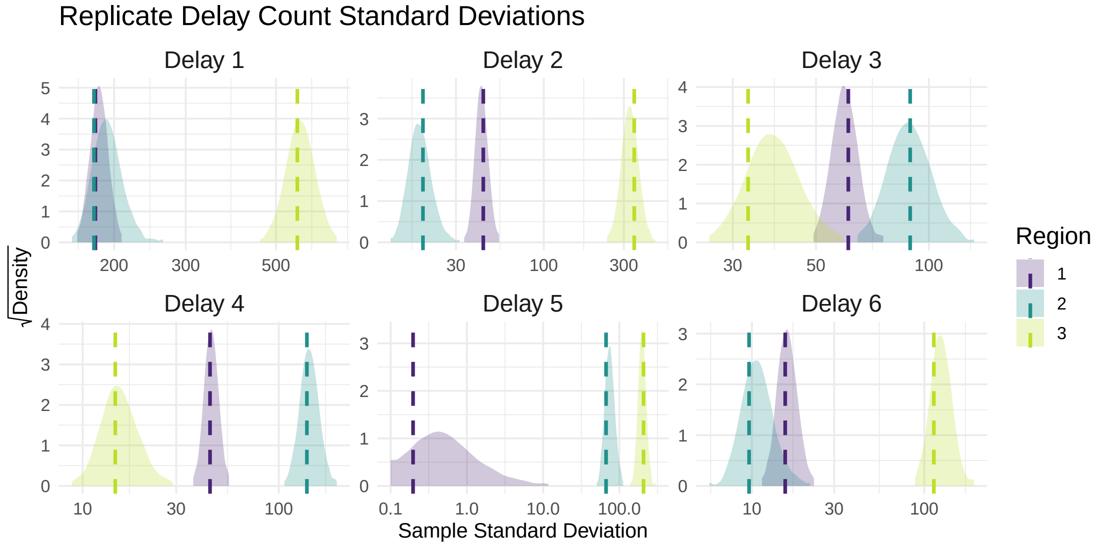

Figure 13: Density estimates of the posterior predictive sample standard deviations of the non-GDM delay counts $(z^{(2)}_{t,s,d})$, for the first 6 delays and each of the 3 regions. Dashed lines show the true values. A log-10 transformation was used for the x-axis, and a square root transformation was used for the y-axis.

We also tested the fit of the GDM to delay counts $z^{(2)}_{t,s,d}$ simulated from a different model, in this case a Binomial-Gaussian mixture. Compellingly, the flexibility of the GDM meant that it was able to capture the variance of these alternative counts very closely.

Appendix D Details of Competing Models for COVID-19 Deaths

In Section 4.5 of the main article, we compare two versions of the GDM against four competing models for COVID-19 deaths in a rolling nowcasting experiment. Here we provide details on the specification and implementation of each of these models.

D.1 GDM Hazard model

With the first version of the GDM being the GDM Survivor model described in Section 4.2, we detail second version based on the Hazard variant of the GDM here. The hierarchical structure is the same as before:

$$y_{t,s} \mid \lambda_{t,s}, \theta_s \sim \text{Negative-Binomial}(\lambda_{t,s}, \theta_s); \quad (11)$$

$$\boldsymbol{z}_{t,s} \mid y_{t,s}, \boldsymbol{\nu}_{t,s}, \boldsymbol{\phi}_{t,s} \sim \text{GDM}(\boldsymbol{\nu}_{t,s}, \boldsymbol{\phi}_{t,s}, y_{t,s}). \quad (12)$$

The model for the mean daily fatality rate $\lambda_{t,s}$ is also the same as before:

$$\log(\lambda_{t,s}) = \iota_s + \delta_{t,s}, \quad (13)$$

with $\delta_{t,s}$ being independent penalised splines of time for each region s, for the purpose of the prediction experiment (as explained in Section 4.5). The difference in the Hazard variant is that the Beta-Binomial means $\nu_{t,s,d}$ are modelled directly:

$$\log\left(\frac{\nu_{t,s,d}}{1-\nu_{t,s,d}}\right) = \psi_{s,d} + \beta_{t,s,d} + \gamma_{t,s,d}. \quad (14)$$

Here, $\psi_{s,d}$ are intercept effects with i.i.d. Normal prior distributions. Then, $\beta_{t,s,d}$ are penalised splines of time and $\gamma_{t,s,d}$ are penalised splines of the day of the week, both independent for each delay d and region s. With different splines of time and weekly cycles for each delay modelled ($d = 1, \ldots, D$), this GDM Hazard model can potentially capture more complex changes in the reporting delay structure over time and more complex weekly cycles than the GDM Survivor model in Section 4.2.

D.2 Negative-Binomial Survivor model (NB Survivor)

The first model competing against the GDM is a Negative-Binomial model intended to approximate the (as-yet) unknown marginal model for the delayed counts $z_{t,s,d}$ obtained from the GDM when integrating out the total counts $y_{t,s}$. The key

feature of this model is that the systematic models for the total count and the reporting delay are otherwise identical to those presented in Section 4.2:

$$z_{t,s,d} \mid \mu_{t,s,d}, \lambda_{t,s}, \theta_s \sim \text{Negative-Binomial}(\mu_{t,s,d}\lambda_{t,s}, \theta_s); \quad (15)$$

$$\log(\lambda_{t,s}) = \iota_s + \delta_{t,s}; \quad (16)$$

where $\mu_{t,s,d}$ is the mean proportion at each delay, again defined by a probit model for the cumulative proportion reported:

$$\mu_{t,s,d} = S_{t,s,d} - S_{t,s,d-1}; \quad (17)$$

$$\text{probit}(S_{t,s,d}) = \psi_{s,d} + \beta_{t,s} + \gamma_{t,s}, \quad (18)$$

and $\lambda_{t,s}$ is the mean of $y_{t,s}$. Like in the GDM model for COVID-19 deaths, we include models for the first $D' = 6$ delayed counts $z_{t,s,d}$, but also an extra (identical) model for the remainder $r_{t,s} = y_{t,s} - \sum_{d=1}^{D'} z_{t,s,d}$ – which is unobserved when $y_{t,s}$ is unobserved (see Stoner and Economou (2020) for the rationale behind modelling the remainder). The model is implemented using MCMC following the specification in Section 4.3 for prior distributions and implementation.

D.3 Negative-Binomial model based on Bastos et al. (INLA)

The second competitor is a Negative-Binomial model for the delay counts $z_{t,s,d}$ based on the framework and implementation detailed in Bastos et al. (2019):

$$z_{t,s,d} \mid \lambda_{t,s,d}, \theta_s \sim \text{Negative-Binomial}(\lambda_{t,s}, \theta_s); \quad (19)$$

$$\log(\lambda_{t,s,d}) = \iota_s + \delta_{t,s} + \beta_{d,s} + \gamma_{t,s,d} + \xi_{t,s,d}. \quad (20)$$

Independently for each region, ι_s are the intercepts, $\delta_{t,s}$ are first order random walks (RW1) (to capture the epidemic curve), $\beta_{d,s}$ are RW1 effects to capture the mean delay distribution, $\gamma_{t,s,d}$ are RW1 temporal effects for each delay to capture temporal variation in the delay distribution, and $\xi_{t,s,d}$ are cyclic RW2 effects of

day of the week for each delay, to capture weekly cycles in the reporting delay. The main difference between this model and the above marginal approximation to the GDM is the lack of separation of systematic variability in the total count and the reporting delay. The model is implemented using the Integrated Nested Laplace Approximation method and the `r-inla` package (Lindgren and Rue, 2015), using code adapted from Bastos et al. (2019).We found that modelling each of the $d = 1, \ldots, 14$ delays separately resulted in extreme 95% prediction interval upper bounds for some data cutoffs (C). We therefore opted to model only the first $D = 6$ delays, together with a remainder term $r_{t,s}$ (as in the NB Survivor model), which solved the problem.

D.4 Models based on McGough et al. (NobBS and NobBS-14)

The third and fourth competitor models are based on the framework for the delayed counts $z_{t,s,d}$ proposed by McGough et al. (2020) and implemented using the `NobBS` package for R. The package allows for both Poisson and Negative-Binomial models, and we opted for the latter to account for over-dispersion:

$$z_{t,s,d} \mid \lambda_{t,s,d}, \theta_s \sim \text{Negative-Binomial}(\lambda_{t,s,d}, \theta_s); \quad (21)$$

$$\log(\lambda_{t,s,d}) = \alpha_{t,s} + \log(\beta_{d,s}); \quad (22)$$

where $\alpha_{t,s}$ is an RW1 effect to capture the epidemic curve and $\beta_{d,s}$ are vectors of proportions such that $\sum_{d=1}^{D_{max}} \beta_{d,s} = 1$ (recall the maximum delay $D_{max} = 14$). Effects $\beta_{d,s}$ therefore capture the expected proportion reported at each delay and are modelled as fixed in time. To account for temporal heterogeneity in the delay distribution, McGough et al. (2020) proposes fitting the model to data in a moving window of fixed length, so that the estimated $\beta_{d,s}$ are more appropriate for recent data. We fit two models using this approach, one with the same 70

day window length as we used for the other approaches (NobBS) and one with a shorter window of 14 days (NobBS-14), so that the estimated mean delay distribution is representative of the last two weeks. In both cases, we used the default weakly-informative prior distributions from the `NobBS` package, which implements the model using the JAGS software facility for MCMC (Plummer, 2003).

D.5 Measurement of computation times

To compare the practicality of each approach, we measured computation times needed to run the whole experiment (all 153 cutoff dates), but some care is needed in how these are interpreted because we did not employ the same parallel computing strategies for each method. All models were run on the same Ubuntu Linux Desktop with an Intel Core i9-7900X CPU and 128Gb of system memory (plus a 256Gb swap drive). For the GDM Survivor, GDM Hazard, and NB Survivor approaches, we ran models (with data for all regions) for different cutoff dates simultaneously across 16 CPU threads. We therefore divide the total run time for these by the ceiling function of (153/16). For the INLA models, we ran models sequentially for each region and for each cutoff date, with parallelisation handled automatically by INLA. We therefore divided the total run time for the INLA approach by 153. Finally, for the NobBS models we ran models for each region in parallel across 7 threads (one per region) and for each cutoff date sequentially. As all the models we tested were independent across regions, they all could feasibly have been run in parallel across regions in the same manner as we did for NobBS. Therefore, for a fair comparison of the relative practicality of each model for daily operation use, we divided the total run time for the NobBS models by (153/7). The resulting indicative run times are presented in the rightmost column of Table 1 in the main article.

Appendix E Moving Data Windows

As surveillance of a disease persists, available data may eventually span months or years. Fitting complex models like those described in this article to long data time series can prove burdensome. For example, when models involve splines of time, as time series grow it may become necessary to increase the number of knots and therefore the number of coefficients to be estimated. It is therefore increasingly common to see models for correcting delayed reporting trained against only the most recent data, falling within a specified moving data "window". For example, Kline et al. (2022) used a moving data window of 90 days to reduce computation time when applying the GDM approach to COVID-19 cases in Ohio. Moving windows also have the potential to improve prediction performance in methods where the delay distribution is assumed constant in time, e.g. McGough et al. (2020), as estimation is only informed by more recent data, which may be more representative of the prediction period than older data.

Here we define a moving window in terms of the date of death, t, and the most recent date of death for which data is available (called the data cutoff in the main article), C. Given a moving window size W (days), we only fit models to partial count data $z_{t,s,d}$ and total count data $y_{t,s}$ falling within the range $C - W + 1 \leq t \leq C$.

All the models in the prediction experiment, unless otherwise indicated, were fitted using a window size of 10 weeks ($W = 70$ days). While we don't aim to fully investigate the optimal choice of W here – which is likely to depend on the method and application – we repeated the rolling prediction experiment in Section 4.5, testing 3 different values of W, to potentially uncover any general patterns in prediction performance and computation time. Taking the GDM Survivor model described in Section 4.2 of the main article, we compared the existing results with $W = 70$ against results from 2 further window sizes, 5 weeks ($W = 35$) and 15

weeks ($W = 105$). To account for the change in the length of the time series, we decreased the number of knots in the splines of time proportionally from 10 to 5 in the models with $W = 35$ (days), and increased the number of knots proportionally to 15 for $W = 105$.

To investigate differences in accuracy, precision, and reliability, we computed mean average errors of predictions, mean 95% prediction interval widths, and 95% prediction interval coverage values. Figure 14 presents these metrics for different prediction time differences. Recall positive time differences indicate forecasting and negative time differences indicate prediction for dates of death in the past, relative to the model fit. The mean average errors are virtually identical across all 3 moving window sizes, meaning a moving window size of 5 weeks ($W = 35$) would be sufficient for accurate point estimates. On the other hand, while window sizes of 10 and 15 weeks had identical mean prediction interval widths, predictions with a 5 week window had substantially greater widths. This means predictions from the model with a 5 week window were more uncertain, especially when forecasting. For all window sizes, 95% prediction interval coverages were consistently above 0.95.

The average computation times were 30 minutes per day with a 5 week window ($W = 35$), 75 minutes with a 10 week window ($W = 70$), and 155 minutes with a 15 week window. The computation time therefore more than doubled in both increments of 5 weeks to the window size. This makes sense, since both the number of data points in the model and the number of spline coefficients to estimate increase as W increases.

In conclusion, we believe a moving window size of $W = 70$ is a reasonable choice for this data, as increasing the window size substantially increased daily computation time with no clear gains in prediction accuracy or precision. Smaller values of W could be tested to identify a window size with similar precision and

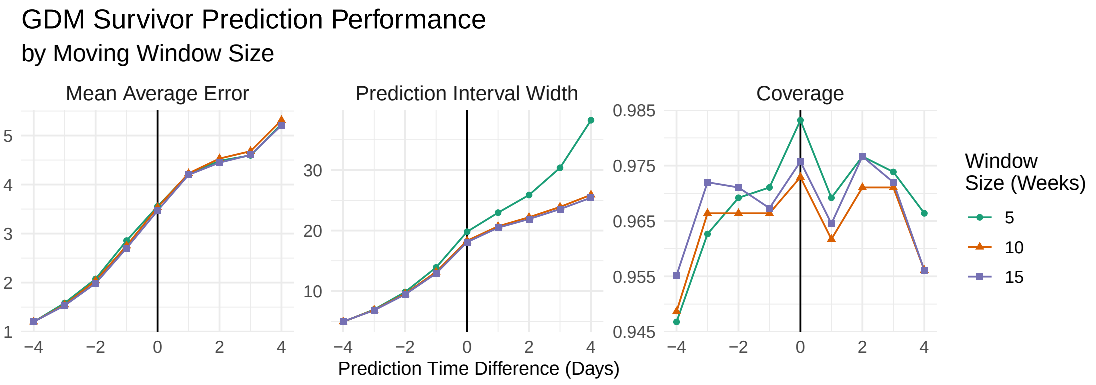

Figure 14: Mean average errors (left), mean 95% prediction interval widths (centre), and 95% prediction interval coverage values (right) for predicted daily COVID-19 deaths from GDM Survivor models with different moving window sizes (weeks). Performance metrics are arranged on the x-axis by prediction time difference, from -4 days up to +4 days, and different moving window sizes are represented by different colours and shapes.

even faster computation times.

Appendix F Under-reporting

Where count data are affected by delayed-reporting, the total reported count, $y_{t,s,d}$, is often still a substantial under-representation of the true count, termed here $x_{t,s,d}$. Reports of COVID-19 cases may, for instance, be affected by under-reporting due to a lack of testing availability, false negative test results, or a lack of symptoms. Similarly, some deaths due to COVID-19 may be missed if the patient was not tested and COVID-19 was not specified on the death certificate. To take this into account, Stoner and Economou (2020) presents a comprehensive framework for simultaneously modelling under-reporting and delayed-reporting. Extended here to include a spatial dimension, this is achieved by replacing Equation (1) in Section

3 of the main text with:

$$
\begin{aligned}
x_{t,s} \mid \lambda_{t,s}, \theta_s &\sim \text{Negative-Binomial}(\lambda_{t,s}, \theta_s); && (23)\\
y_{t,s} \mid x_{t,s}, \pi_{t,s} &\sim \text{Binomial}(\pi_{t,s}, x_{t,s}); && (24)\\
\log\left(\frac{\pi_{t,s}}{1-\pi_{t,s}}\right) &= i(t,s), && (25)
\end{aligned}
$$

for $y_{t,s} \leq x_{t,s}$ and where $i(t,s)$ is a general function which may include covariates or random effects, e.g. covariates representing access to COVID-19 tests. The likelihood for $y_{t,s}$ is non-identifiable between a high $\lambda_{t,s}$ and a low $\pi_{t,s}$, or vice-versa, so in the case where all available counts are assumed potentially under-reported (i.e. $x_{t,s}$ is always unobserved), identifiability can be achieved using prior information (Stoner et al., 2019).

References

Bastos, L. S., T. Economou, M. F. C. Gomes, D. A. M. Villela, F. C. Coelho, O. G. Cruz, O. Stoner, T. Bailey, and C. T. Codeço (2019). A modelling approach for correcting reporting delays in disease surveillance data. Statistics in Medicine 38(22), 4363–4377.

Codeço, C. T., J. d. S. Cordeiro, A. W. d. S. Lima, R. A. Colpo, O. G. Cruz, F. C. Coelho, P. M. Luz, C. J. Struchiner, and F. R. d. Barros (2012). The epidemic wave of influenza A (H1N1) in Brazil, 2009. Cadernos de saúde pública 28(7), 1325–1336.

de Valpine, P., D. Turek, C. J. Paciorek, C. Anderson-Bergman, D. T. Lang, and R. Bodik (2017). Programming with models: Writing statistical algorithms for general model structures with NIMBLE. Journal of Computational and Graphical Statistics 26(2), 403–413.

Gelman, A., J. Carlin, H. Stern, D. Dunson, A. Vehtari, and D. Rubin (2014, November). Bayesian Data Analysis, Third Edition (Chapman and Hall/CRC Texts in Statistical Science) (Third ed.). London: Chapman and Hall/CRC.

Kline, D., A. Hyder, E. Liu, M. Rayo, S. Malloy, and E. Root (2022, 02). A Bayesian Spatiotemporal Nowcasting Model for Public Health Decision-Making and Surveillance. American Journal of Epidemiology 191(6), 1107–1115.

Lindgren, F. and H. Rue (2015). Bayesian spatial modelling with r-inla. Journal of Statistical Software, Articles 63(19), 1–25.

McGough, S. F., M. A. Johansson, M. Lipsitch, and N. A. Menzies (2020, 04). Nowcasting by bayesian smoothing: A flexible, generalizable model for real-time epidemic tracking. PLOS Computational Biology 16(4), 1–20.

Plummer, M. (2003). Jags: A program for analysis of bayesian graphical models using gibbs sampling.

Stoner, O. and T. Economou (2020). Multivariate hierarchical frameworks for modeling delayed reporting in count data. Biometrics 76(3), 789–798.

Stoner, O., T. Economou, and G. Drummond Marques da Silva (2019). A hierarchical framework for correcting under-reporting in count data. Journal of the American Statistical Association.

World Health Oganization (2014). WHO surveillance case definitions for ILI and SARI. `https://www.who.int/influenza/surveillance_monitoring/ili_sari_surveillance_case_definition/en/`. Accessed 2019-09-25.
